# Genetically encoded multimeric tags for subcellular protein localization in cryo-EM

**DOI:** 10.1038/s41592-023-02053-0

**Published:** 2023-11-06

**Authors:** Herman K. H. Fung, Yuki Hayashi, Veijo T. Salo, Anastasiia Babenko, Ievgeniia Zagoriy, Andreas Brunner, Jan Ellenberg, Christoph W. Müller, Sara Cuylen-Haering, Julia Mahamid

**Affiliations:** 1 Structural and Computational Biology Unit, European Molecular Biology Laboratory (EMBL), Heidelberg, Germany; 2 Cell Biology and Biophysics Unit, European Molecular Biology Laboratory, Heidelberg, Germany; 3 University of Heidelberg, Heidelberg, Germany; 4 Faculty of Biosciences, Collaboration for Joint PhD Degree between EMBL and Heidelberg University, Heidelberg, Germany

**Keywords:** Cryoelectron microscopy, Cryoelectron tomography, Cell biology, Biophysics

## Abstract

Cryo-electron tomography (cryo-ET) allows for label-free high-resolution imaging of macromolecular assemblies in their native cellular context. However, the localization of macromolecules of interest in tomographic volumes can be challenging. Here we present a ligand-inducible labeling strategy for intracellular proteins based on fluorescent, 25-nm-sized, genetically encoded multimeric particles (GEMs). The particles exhibit recognizable structural signatures, enabling their automated detection in cryo-ET data by convolutional neural networks. The coupling of GEMs to green fluorescent protein-tagged macromolecules of interest is triggered by addition of a small-molecule ligand, allowing for time-controlled labeling to minimize disturbance to native protein function. We demonstrate the applicability of GEMs for subcellular-level localization of endogenous and overexpressed proteins across different organelles in human cells using cryo-correlative fluorescence and cryo-ET imaging. We describe means for quantifying labeling specificity and efficiency, and for systematic optimization for rare and abundant protein targets, with emphasis on assessing the potential effects of labeling on protein function.

## Abstract (web summary)

Genetically encoded multimeric particles (GEMs) are 25-nm tags with recognizable structural signatures, which can be used to label specific proteins in mammalian cells to facilitate their subcellular localization in cryo-ET.

## Main

Cryo-electron tomography (cryo-ET) has emerged as a powerful label-free method for visualizing and quantitatively analyzing subcellular architectures, structures of large and abundant macromolecular complexes, and their context-dependent interactions in intact cells^1–5^. However, the localization and identification of specific structures of interest in the crowded intracellular landscapes visualized by cryo-ET are challenging tasks. Present solutions include: (1) cryo-correlative light and electron microscopy (CLEM)^6,7^, whereby fluorescence is used to guide cryo-ET sample preparation^8^, image acquisition^9^ and interpretation, albeit with localization errors due to limited resolution in fluorescence imaging and sample deformation during preparation or transfers^8^; (2) computational pattern recognition approaches, based on template matching^10^ or convolutional neural networks (CNNs)^11,12^, which are applicable to large molecular assemblies with known or identifiable structures; and (3) molecular tags with unique sizes, shapes or densities, which have been proposed as a complementary solution for direct molecular localization in cryo-electron tomograms. However, available tags based on iron-enriching ferritin^13,14^ and DNA origami^15^ have limited applicability inside mammalian cells. In current implementations of ferritin as a fusion tag^13^ or the ligand-inducible FerriTag^14^, soluble iron is applied to cells for chelation by ferritin, thereby enhancing contrast. Exogenous iron can be cytotoxic to mammalian cells, and while omittable, the 12-nm ferritin assembly without iron can be difficult to distinguish from other globular macromolecular species in cellular cryo-ET. DNA origami assemblies such as SPOTs^15^, which harbor a green fluorescent protein (GFP)-binding RNA aptamers for labeling of cell surface and extracellular GFP-tagged proteins, require folding in vitro and can be difficult to introduce into cells without physical or chemical disruption. To address these limitations of available tags for intracellular protein labeling in mammalian cells, we present the development of an alternate strategy for subcellular-level localization of structures of interest in cryo-ET.

**Fig. 1:**
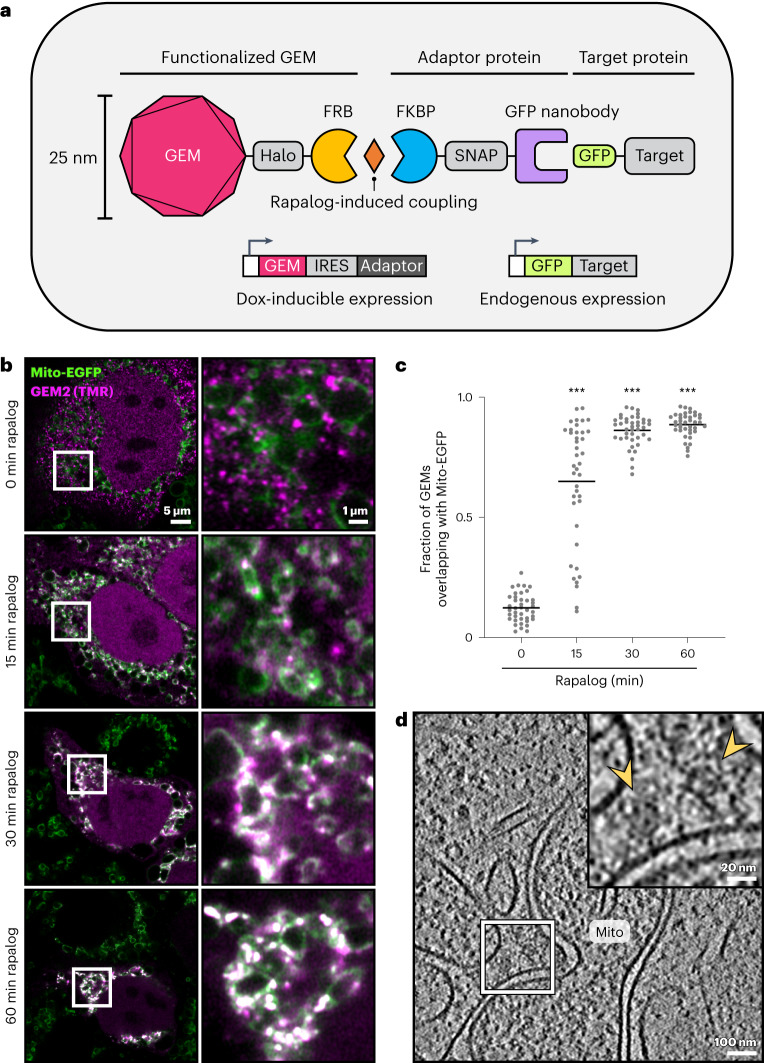
GEM2 labeling of mitochondrial surface-displayed EGFP in human cells. **a**, Schematic of the labeling system. **b**, Time course of GEM2 (fluorescently labeled with TMR, magenta) recruitment to Mito-EGFP (green) upon rapalog treatment by fluorescence microscopy in HeLa cells. GEM2 and adaptor protein were expressed from the AAVS1 locus (stable knock-in) with 24–48 h doxycycline induction before rapalog treatment. **c**, Quantification of **b**, showing the fraction of GEMs overlapping with Mito-EGFP per cell. Lines indicate mean, *n* (left to right) = 40, 40, 41, 40 cells, two experiments. ***P < 0.0001, Kruskall–Wallis test followed by Dunn’s test, compared to 0 min. **d**, Tomographic slice showing a GEM2-labeled mitochondrion after 30 min rapalog treatment. Arrowheads in the inset indicate GEM2 particles. Source data

Our design concept encompasses a genetically encoded tag that is structurally distinct in the cell, is recognizable in cryo-electron tomograms and that tethers to GFP on addition of a small molecule, thereby enabling time-controlled labeling of GFP-fusion proteins (Fig. 1a). To achieve a distinct structural signature, the tag is based on an encapsulin protein scaffold. Native to archaea and bacteria, encapsulins assemble into icosahedral particles of distinct stoichiometries, with triangular numbers *T* = 1, 3 and 4, ranging from 60 to 240 subunits, and 25 to 42 nm in size^16^. *T* = 3 encapsulins have been shown to self-assemble in mammalian cells and exhibit a distinct appearance in cryo-electron tomograms^17,18^. It was shown recently that the *T* = 4 *Quasibacillus thermotolerans*, *T* = 3 *Myxococcus xanthus* and *T* = 1 *Thermotoga maritima* encapsulins can be engineered with heavy metal-chelating elements and nanobodies for intracellular labeling in room-temperature electron microscopy (EM)^19^. Thus, encapsulin-derived genetically encoded multimeric particles (GEMs) appear to be a suitable platform for the development of an intracellular cryo-EM tag. The size and stoichiometry of the tag is likely to influence its diffusion and impact on target protein function in cells^17^. Therefore, the smallest of encapsulins (*T* = 1, 25 nm) represent promising scaffolds that can at the same time provide an adequate size for visual detection in cryo-ET without the need for heavy metal enrichment. To reduce the impact of the multimeric tag on native protein function, we adopt a ligand-controlled coupling strategy as used in FerriTag^14^. By decorating the surface of the encapsulin with the FKBP-rapamycin-binding (FRB) domain of mTOR, we enable inducible coupling to GFP via an adaptor protein, consisting of the FKBP and an anti-GFP nanobody^20^, upon addition of a rapamycin analog before sample vitrification (Fig. 1a). By targeting GFP, the system is aimed to be applicable to the wide range of available GFP-tagged cell line and plasmid resources. Finally, to enable systematic assessment and optimization of labeling efficiency for different subcellular structures, we incorporate into the encapsulin and adaptor constructs Halo- and SNAP-tags for fluorescent imaging. Here, we detail the identification of a suitable GEM scaffold and demonstrate the application of our developed tag for the labeling and subcellular localization of a number of endogenous and overexpressed targets across different locations in human cell lines.

## Results

### Identifying suitable encapsulins for ligand-induced coupling

**Extended Data Fig. 1:**
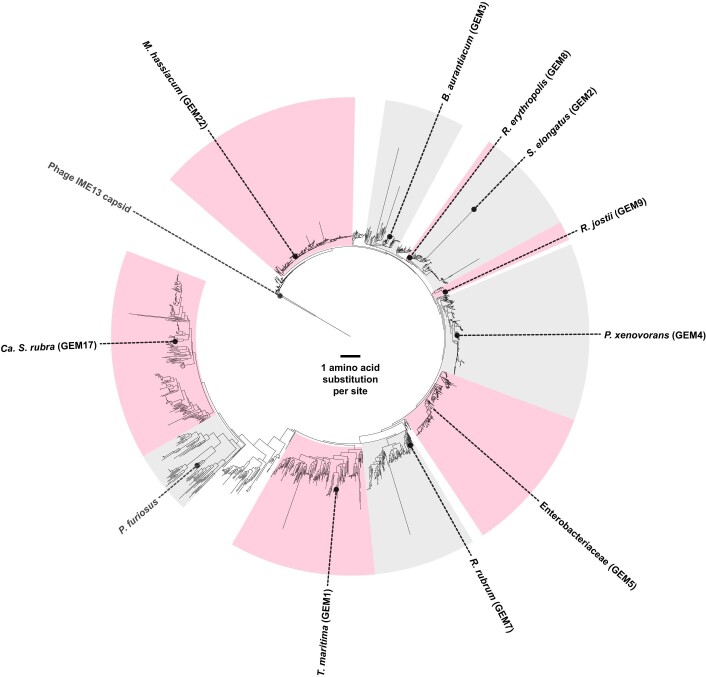
Phylogenetic diversity of selected encapsulins. Maximum likelihood tree of Family 1 encapsulins as identified and constructed by Andreas et al.^16^, with Family 2 A encapsulin *Synechococcus elongatus* SrpI (GEM2) and *Stenotrophomonas* phage IME13 capsid protein (outgroup). Scale bar represents amino acid substitution per site. Indicated encapsulins, with GEM IDs in brackets, have been shown to form 25-nm-sized *T* = 1 particles, detailed in Supplementary Table 1. GEM1 has been engineered with heavy-metal-chelating elements and surface nanobodies (EMcapsulin) for room-temperature EM localization^19^. Also indicated is the *T* = 3 particle-forming encapsulin of *Pyrococcus furiosus*, previously used in budding yeast and HEK293 cells as a rheology probe^17^. Clades are shaded in alternating colours up to the most recent common ancestor between annotated sequences.

**Extended Data Fig. 2:**
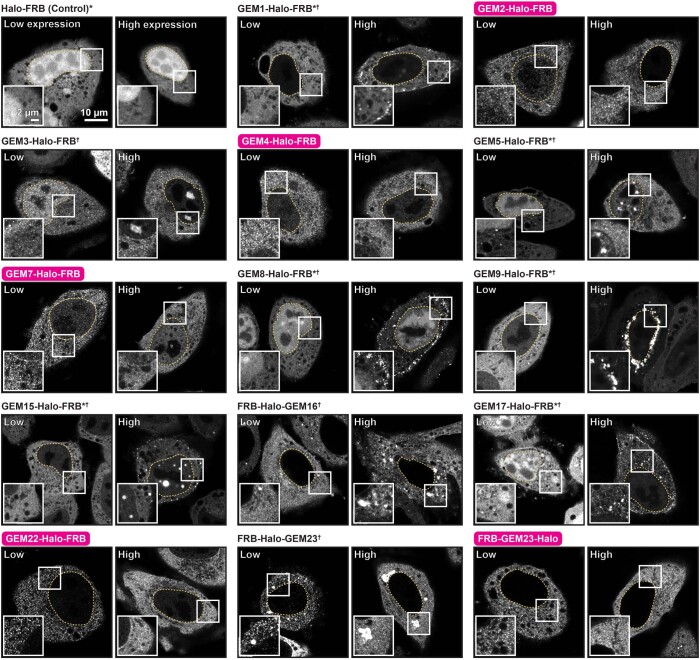
GEM-Halo-FRB fusion expression screen. Constructs were transiently expressed in HeLa cells under a cytomegalovirus (CMV) promoter and labeled with Halo-TMR. The transfections resulted in a range of GEM expression levels. Boxed labels in pink indicate constructs that give rise to predominantly uniformly sized fluorescent puncta. Asterisks indicate constructs with soluble protein localisation. Daggers indicate aggregation, which was more prominent at high expression levels for some constructs. Dashed lines mark the cell nucleus based on Hoechst staining imaged in a separate channel. Low expression examples are displayed with identical contrast settings with respect to one another. High expression examples from the same experiment, defined here as cells with five times brighter fluorescence, are displayed with identical contrast settings. Representative images, experiment performed twice with similar results.

**Extended Data Fig. 3:**
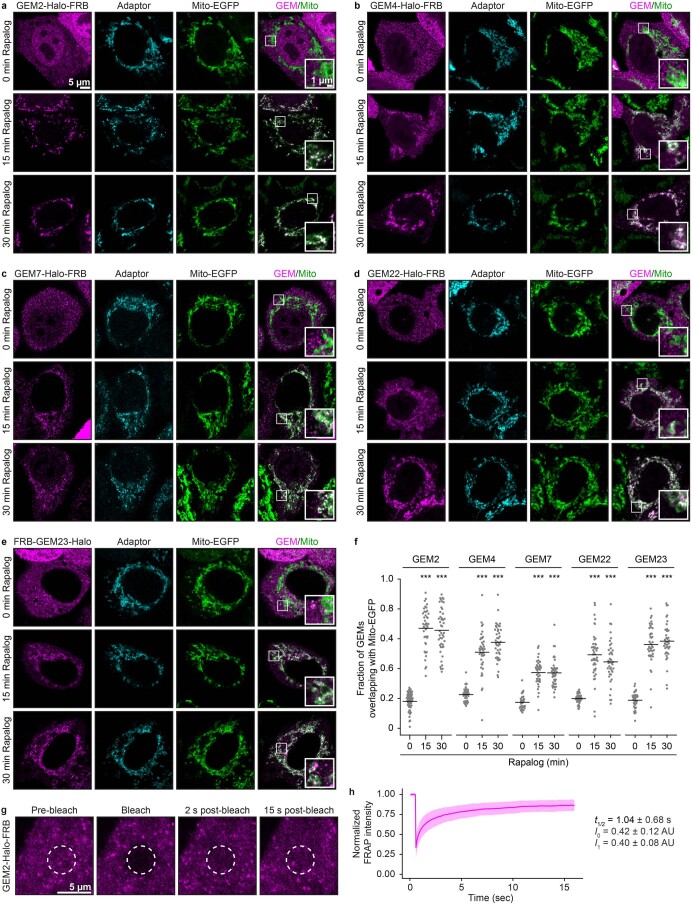
Mito-EGFP GEM coupling screen. HeLa cells stably expressing Mito-EGFP were transiently transfected with **a**, GEM2/adaptor; **b**, GEM4/adaptor; **c**, GEM7/adaptor; **d**, GEM22/adaptor; or **e**, GEM23/adaptor. Upon 24 h doxycycline induction, GEMs and adaptor proteins were labelled with Halo-TMR and SNAP-SiR, respectively. Cells were treated with rapalog for indicated time points. **f**, Fraction of GEMs overlapping with Mito-EGFP per cell. Lines indicate mean. Number of cells analysed per group (left to right): *n* = 52, 42, 46, 38, 41, 42, 32, 37, 41, 39, 46, 41, 33, 40, 40, 3 experiments. ***P < 0.0001, Kruskall-Wallis test followed by Dunn’s test, compared to 0 min treatment. **g**, GEM2 fluorescence recovery after photobleaching (FRAP) assay. Fluorescence images of GEMs in HeLa cells stably expressing Mito-EGFP with GEM2/adaptor knock-in before and after photobleaching, in the absence of rapalog. GEMs were labelled with TMR. Dashed circle indicates the photobleached region. **h**, GEM2 fluorescence recovery curves. Magenta indicates mean (solid line) ± s.d. (shaded area). Single-exponential curves were fitted to individual recovery curves with *t*_1/2_, *I*_0_, and *I*_1_ representing the recovery half-life, normalised intensity immediately post-bleach, and the dynamic range of recovery, respectively. Analysis of g, *n* = 90 cells, 3 experiments. Source data

**Extended Data Fig. 4:**
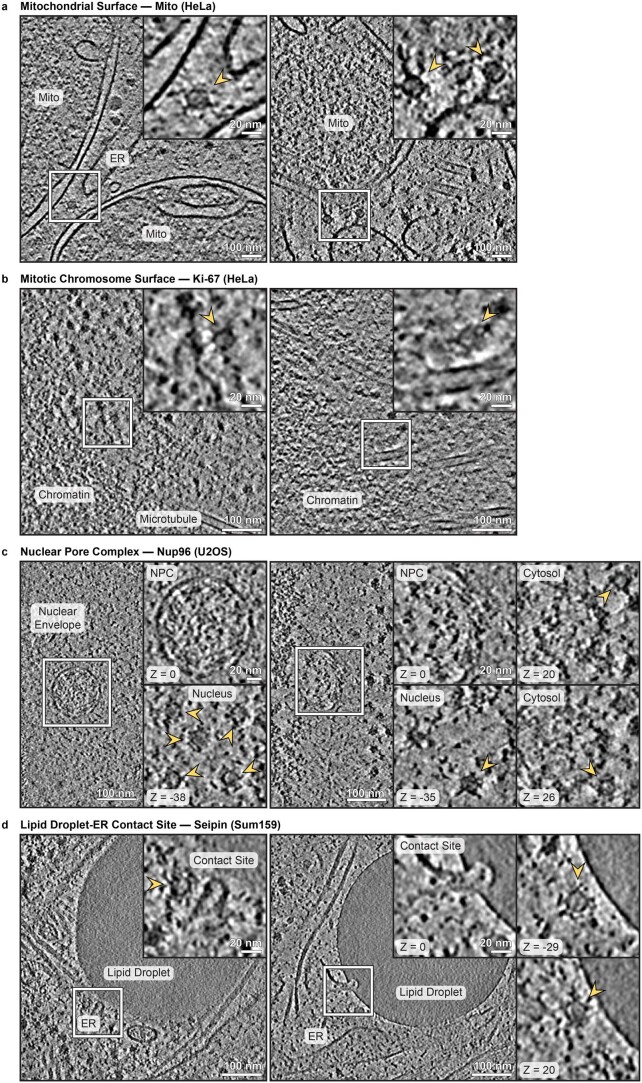
Additional examples of GEM2 localisation at the subcellular targets by cryo-ET. **a**, Overexpressed Mito-EGFP. **b**, Endogenous Ki-67. **c**, Endogenous Nup96. **d**, Endogenous seipin. Each example is taken from a different cell. Arrowheads indicate GEM particles in enlarged insets. Particles labelling the same structure on different z-slices are shown for Nup96 and seipin. Slice numbers indicated are in steps of 1.37 nm for Nup96 and 1.35 nm for seipin, respectively. At some nuclear pore complexes (NPCs), GEMs are observed near both cytoplasmic and nuclear rings, where Nup96 localises.

The functionalization of encapsulin surfaces beyond the addition of peptide tags is not trivial^21^ as it can disrupt critical contacts required for particle assembly^19,22^. Therefore, to identify constructs that are compatible with our design (ligand-inducible coupling via FRB-FKBP and fusion with a fluorescent reporter), we conducted an expression screen of ten naturally occurring encapsulins^23–31^ and three synthetic cages^32–34^ in HeLa cells, all expected to form 25-nm-sized, *T* = 1 icosahedral particles (Extended Data Fig. 1 and Supplementary Table 1). Fluorescence imaging of Halo-FRB-tagged constructs revealed five candidates that yield uniformly sized puncta in the cytoplasm, suggesting that the expressed proteins can assemble into discrete particles (Extended Data Fig. 2). Among them, three GEMs, including the functionalized *Synechococcus elongatus* Srp1 encapsulin (GEM2), also localize to the nucleus. Next, we evaluated the five candidates’ potential for coupling to GFP on treatment with a relatively inert analog of rapamycin, rapalog AP21967 (ref. ^35^), in cells that stably express enhanced green fluorescent protein (EGFP) on the mitochondrial surface as a test case. The functionalized GEMs and adaptor protein were introduced via a single doxycycline-inducible gene cassette (Fig. 1a). We found that GEM2 colocalized with mitochondrial-targeted EGFP (Mito-EGFP) most efficiently (Extended Data Fig. 3a–f) and could be recruited to Mito-EGFP within 15 min on treatment with rapamycin or rapalog (Fig. 1b,c and Supplementary Video 1). We further confirmed that GEM2 was mobile in the cytoplasm, as indicated by time-lapse imaging (Supplementary Video 2) and a fluorescence recovery after photobleaching half-life of 1.0 ± 0.7 s (Extended Data Fig. 3g,h). In agreement with these observations, cryo-ET imaging of focused ion beam (FIB) lamellae from cells after 30 min of rapalog treatment revealed 25-nm-sized icosahedral particles close to the mitochondrial surface (Fig. 1d and Extended Data Fig. 4a).

### GEM2 labels endogenous targets with differential dynamics

**Fig. 2:**
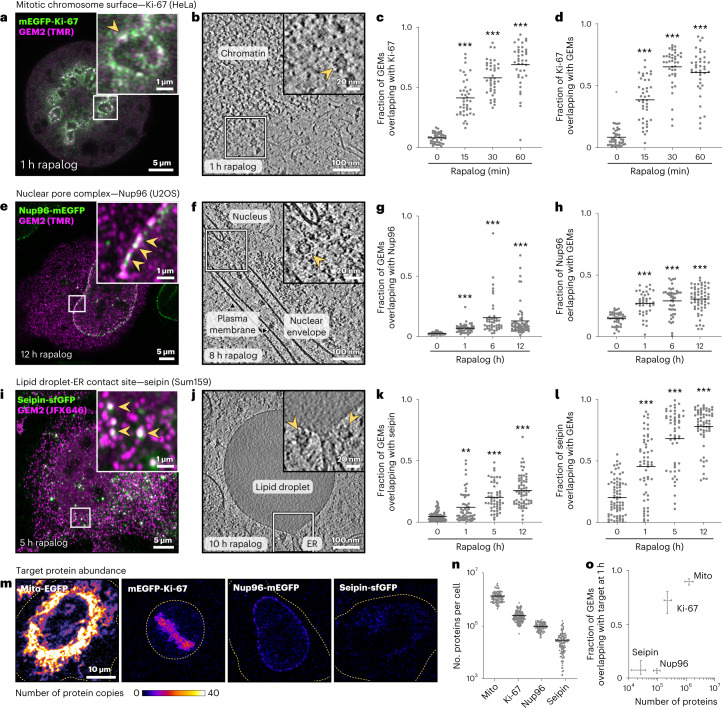
GEM2 labels endogenously GFP-tagged proteins in human cells. **a**–**d**, Ki-67. **e**–**h**, Nup96. **i**–**l**, seipin. Left to right: fluorescence images (**a**,**e**,**i**), arrowheads in enlarged insets indicate examples of colocalization between GEM2 and the target protein. Tomographic slices (**b**,**f**,**j**), arrowheads in enlarged insets indicate GEM particles. The corresponding rapalog treatment time is indicated for each image. GEM2 and adaptor protein were expressed from the AAVS1 locus for Ki-67 and seipin, and transiently expressed for Nup96, by doxycycline treatment for 24–48 h before rapalog treatment. Plotted are the fractions of GEMs overlapping with the target proteins (**c**,**g**,**k**), and target proteins overlapping with GEMs for the same cells (**d**,**h**,**l**), evaluated by light microscopy. Lines indicate the mean. Number of cells analyzed per group: *n* = 41, 41, 40, 39 (Ki-67; **c**,**d**), *n* = 41, 41, 44, 58 (Nup96; **g**,**h**) and *n* = 76, 56, 53, 64 (seipin; **k**,**l**), two experiments. **P = 0.0005. ***P < 0.0001, Kruskall–Wallis test followed by Dunn’s test, compared to 0 h rapalog treatment. **m**, Target protein abundance by FCS-calibrated imaging. Representative image slices colored by calibrated protein numbers. Dashed lines indicate cell boundaries. **n**, Analysis of total cellular protein abundances as determined by FCS-calibrated imaging (**m**) combined with 3D segmentation. Number of cells analyzed per target protein: *n* = 92 (mito-EGFP), 148 (Ki-67), 86 (Nup96), 118 (seipin), two experiments. Lines indicate mean. **o**, Fraction of GEMs overlapping with the target at 1 h rapalog treatment as a function of target protein abundance (median and interquartile range) for each target. Analysis of **c**, **g**, **k** and **n**, number of cells and experiments as indicated above. Source data

**Extended Data Fig. 5:**
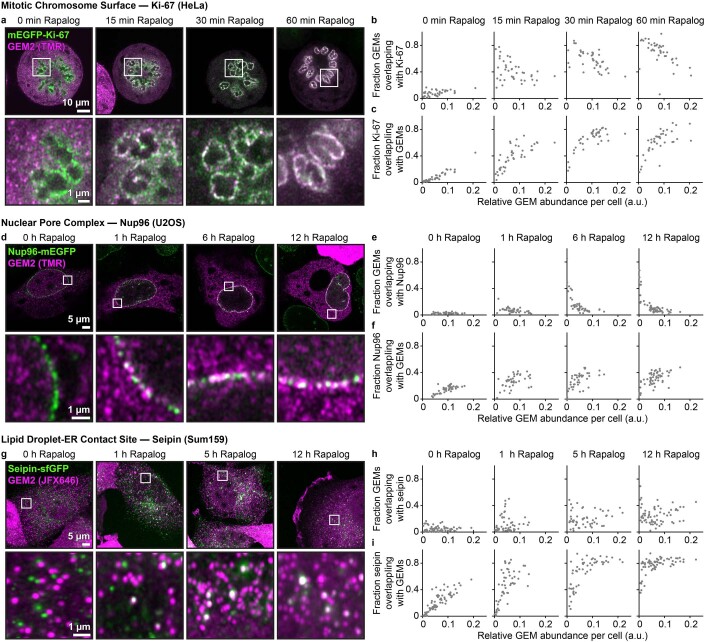
GEM2 recruitment dynamics is dependent on its abundance. **a, d, g**, Time course of GEM2 recruitment to endogenous Ki-67, Nup96 and seipin. GEM2 and adaptor expression from the AAVS1 locus was induced with 24–48 h doxycycline treatment prior to rapalog treatment for the indicated durations. Image for seipin at 5 h is the same as in Fig. 2i, but rotated and with a larger field of view shown. **b**, Fractions of GEMs overlapping with the target as a function of relative GEM abundance, defined as the number of GEM-positive pixels divided by total cellular area per cell. Replotting of data presented in Fig. 2c. Lower GEM abundance gave rise to a higher fraction of GEMs at the target protein. **c**, Fractions of the target overlapping with GEMs as a function of relative GEM abundance per cell. Replotting of data presented in Fig. 2d. Longer rapalog treatment times increased labelling of Ki-67 by GEMs. Higher GEM abundance led to more complete coverage of Ki-67. These results demonstrate the importance of tuning GEM expression levels in the labelling experiment. **e**, Replotting of data in Fig. 2g. **f**, Replotting of data in Fig. 2h. **h**, Replotting of data in Fig. 2k. **i**, Replotting of data in Fig. 2l. Source data

To assess the applicability of GEMs in labeling endogenous human proteins, we targeted GEM2 to three endogenously GFP-tagged proteins at different subcellular locations: Ki-67 at the mitotic chromosome surface^36^, Nup96 at the nuclear pore^37^ and seipin at endoplasmic reticulum-lipid droplet (ER-LD) contact sites^38^. In all cases, we observed rapalog-induced colocalization of GEMs with the target substructure by fluorescence microscopy and cryo-ET (Fig. 2 and Extended Data Fig. 4b–d). We noted that the dynamics and efficiency of GEM recruitment (Fig. 2c,d,g,h,k,l and Extended Data Fig. 5) differed between the targets, and found it to be correlated with the endogenous target protein abundance as measured by fluorescence correlation spectroscopy (FCS) calibrated imaging^39^ (Fig. 2m–o). For the abundant Ki-67, recruitment was rapid and near-complete within 30–60 min of rapalog induction, whereas for the less abundant seipin, recruitment continued to increase after 5 h. Further, while high GEM abundance in the cell favored more complete labeling of the target, it resulted in more GEMs not bound to the target, giving rise to a higher background (Extended Data Fig. 5b,c,e,f,h,i). Conversely, low GEM abundance resulted in higher proportions of GEMs colocalizing with the target. These results highlight the need to optimize GEM expression levels and labeling durations to achieve the desired balance between complete labeling and minimal background for each protein targeted.

**Extended Data Fig. 6:**
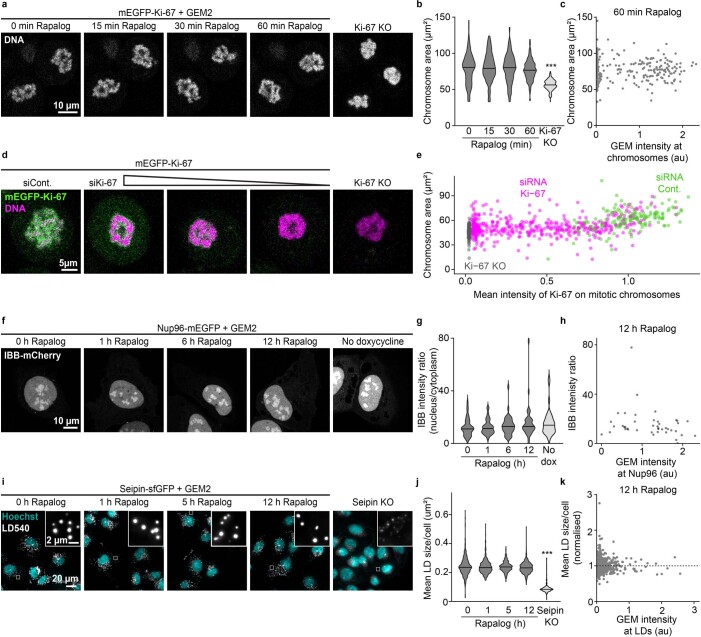
GEM2 recruitment to endogenous Ki-67, Nup96 and seipin has little effect on cellular phenotype. **a**, Mitotic chromosomes (stained with SiR-DNA) after induction of Ki-67 GEM-labelling with rapalog treatment for the indicated time. Ki-67 knock-out (KO) cells serve as a control for aberrant mitotic chromosome coalescence upon Ki-67 impairment^36^. **b**, Mitotic chromosome area measurements in GEM-expressing cells, *n* = 139, 154, 158, 165, 188 cells per treatment (left to right), 2 experiments. Lines indicate median. ***P < 0.0001, Kruskall-Wallis test followed by Dunn’s test, compared to 0 h treatment. **c**, Chromosome area at 60 min treatment as a function of mean GEM fluorescence intensity at chromosomes, *n* = 255 cells, 2 experiments. **d**, Mitotic chromosomes (DNA, magenta) and endogenous mEGFP-Ki-67 signal (green) upon transfection with control siRNA (siCont.) and Ki-67 siRNA (siKi-67) in comparison with Ki-67 KO cells. Cells were transfected with 0.05–5 pmol siRNAs for partial knockdown. **e**, Chromosome area as a function of mean mEGFP-Ki-67 fluorescence intensity on chromosomes. *n* = 156 (siRNA Cont.), 459 (siRNA Ki-67), 150 (Ki-67 KO) cells, 2 experiments. **f**, Importin β binding domain (IBB)-mCherry localization after induction of Nup96 GEM-labelling for the indicated time. Non-doxycycline-induced cells, thus not expressing GEM or adaptor protein, were included as a control. Impairment of nuclear pore integrity results in redistribution of IBB to the cytoplasm^89^. **g**, IBB-mCherry intensity ratio (nucleus/cytoplasm), *n* = 41, 40, 42, 42, 39 cells per treatment (left to right), 2 experiments. Lines indicate median. **h**, IBB-mCherry intensity ratio at 12 h rapalog treatment time as a function of total GEM intensity on Nup96 in the same cells. **i**, Lipid droplets (LDs, stained with LD540) after induction of seipin GEM-labelling for the indicated time. Cells were treated with oleic acid during the final hour of rapalog treatment to induce LD biogenesis. Seipin KO cells serve as a control for mean LD size reduction upon seipin impairment^38^. Contrast is adjusted in insets for comparison with small LDs of seipin KO cells. **j**, Mean LD size per cell, *n* = 594, 610, 639, 738, 335 cells per treatment (left to right), 2 experiments. Lines indicate median. ***P < 0.0001, Kruskall-Wallis test followed by Dunn’s test, compared to 0 h treatment. **k**, Mean LD size at 12 h rapalog treatment time as a function of mean GEM intensity at LDs in the same cells. Dashed line indicates the mean LD size of control cells. Source data

The size (25 nm) and multimeric (60-subunit) nature of GEMs further pose a concern for target protein function due to potential steric hindrance, alteration of hydrodynamics or induction of clustering. While it is challenging to assess the impact of labeling on the level of a single tagged molecule, we observed no changes in the key phenotype associated with each labeled protein target (Extended Data Fig. 6). Specifically, we found no correlation between GEM fluorescence intensities at the target structure and the measured phenotype per cell, even at high GEM expression levels (Extended Data Fig. 6c,h,k). Further, whereas a 20% knockdown in Ki-67 protein level was sufficient to cause aberrant coalescence of mitotic chromosomes, measurable as a decrease in total chromosome area (Extended Data Fig. 6e), we observed no apparent reduction in total mitotic chromosome area on GEM labeling, which in some cells reached as high as 90% efficiency (Fig. 2d and Extended Data Fig. 6c). Altogether, these data indicate that within the timescales investigated, GEM2 labeling of the three endogenous protein targets did not perturb the native cellular phenotype. Nevertheless, careful assessment of protein function and cellular phenotype for each target is recommended during optimization of GEM labeling.

### Quantification of GEM2 labeling by cryo-ET

**Fig. 3:**
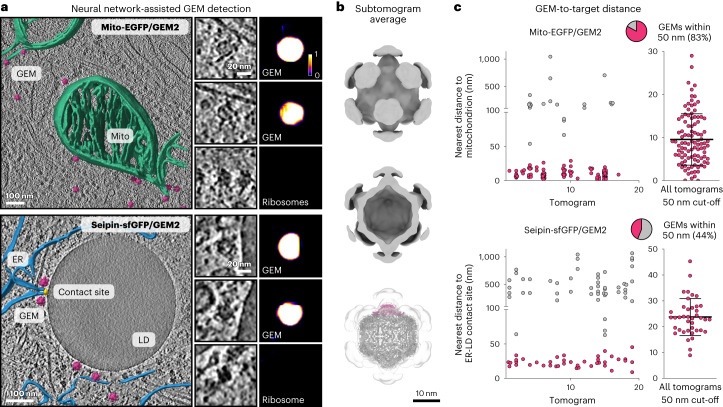
CNN-based detection of GEM-labeled proteins. **a**, Tomographic slices of GEM2-labeled Mito-EGFP on mitochondria (top, also Supplementary Video 3) and seipin-sfGFP near an ER-LD contact site (bottom). Magenta, GEM2 subtomogram averages pasted into the tomogram for visualization; green, mitochondria; blue, ER; yellow, ER-LD contact site. Insets show individual GEMs or ribosomes and corresponding CNN probability scores for each particle. **b**, GEM2 subtomogram average superposed with the structure of the encapsulin scaffold (bottom, PDB 6X8M), with one pentamer represented in magenta. **c**, Spatial distributions of GEMs relative to the outer mitochondrial membrane (top) and ER-LD contact site (bottom) per tomogram, *n* = 123 and 91 GEMs from 17 and 19 tomograms, respectively. Lines indicate mean and s.d. in the right panels. Pie charts indicate the proportion of GEMs within 50 nm of the target subcellular structure (magenta). Source data

**Extended Data Fig. 7:**
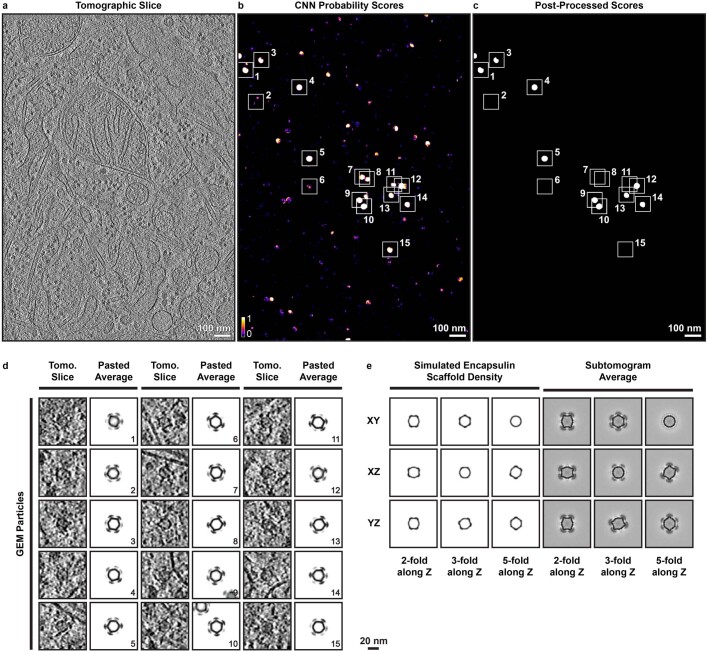
CNN detection and validation of GEM2 particles. **a**, Full view of the tomographic slice from Fig. 3a. **b**, Maximum projection of raw CNN probability scores. **c**, Maximum projection of post-processed CNN probability scores. Scores were thresholded at 0.5, filtered by size (connected-component size cluster of 5000–50000 pixels at 13.7 Å/pixel), and masked with a lamella mask to exclude false positives. This tomogram was not used for CNN training. Numbered boxes correspond to curated particles, shown in d. **d**, Left columns, tomographic slices at the indicated positions in b and c, 6.74 nm in thickness. Right columns, slices through the thresholded subtomogram average pasted in the tomogram based on the refined position and orientation. Of the 15 particles, 9 corresponded to peaks in the post-processed scores, and 6 more (2, 6, 7, 8, 11, 15) were annotated based on visual inspection of lower scoring peaks. **e**, Left, cross-sections of simulated densities at 30 Å resolution based on the *in vitro* structure of the encapsulin scaffold (PDB 6X8M). Right, cross-sections of the GEM2 subtomogram average presented in Fig. 3b.

We have shown that GEM2 can be detected visually based on its unique size and shape at the expected subcellular location corresponding to each protein targeted (Figs. 1 and 2 and Extended Data Fig. 4). To enable unbiased and automated localization of GEMs in cryo-electron tomograms, we trained a CNN^12^ to assist with their identification, based on 1284 particles from 71 tomograms (Fig. 3a, Extended Data Fig. 7 and Supplementary Video 3). Following manual curation of the CNN predictions, where we took into account the appearance of each detected particle and corresponding CNN probability scores (Extended Data Fig. 7b–d), we confirmed by subtomogram averaging that GEM2 forms an icosahedron of the expected size in cells. The subtomogram average superposed well with a previously determined in vitro structure of the *S. elongatus* encapsulin scaffold^24^, and revealed additional densities at the fivefold vertices that correspond to the expected locations of the engineered C-terminal Halo- and FRB-tags (Fig. 3b).

We found that the extent of GEM recruitment to the expected subcellular location correlated with target protein abundance, in line with our light microscopy analyses. In detail, for the abundant Mito-EGFP, 83% of particles localized within 50 nm of the mitochondrial surface, whereas for the less abundant seipin, 44% localized within 50 nm of the ER-LD contact site (Fig. 3c). We measured a distance of 10 ± 6 nm between the encapsulin surface and mitochondrial membrane, and a larger distance of 24 ± 7 nm to the ER-LD contact site, likely due to the positioning of GFP on the extended cytosolic tail of seipin^40^. These values provide an estimate of the precision with which a target can in principle be localized in 3D space using GEMs. However, the symmetric nature of GEM2 and semiflexible linkers incorporated between GEM2 and the target protein prevent unambiguous identification and orientational assignment of the tagged protein molecule solely via GEM2.

**Extended Data Fig. 8:**
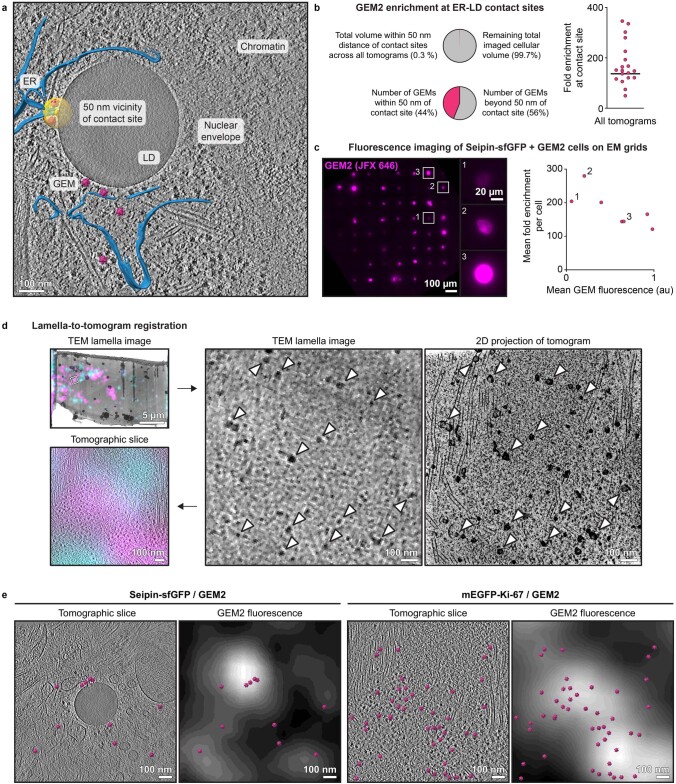
CLEM-based assessment and optimization of GEM2 labelling. **a**, Full view of the tomographic slice from Fig. 3a. Yellow region indicates an ER-LD contact site dilated in all directions by 50 nm and masked with the lamella mask. Lamella masks were defined geometrically based on cross-sections at the front and back of the lamella per tomogram. **b**, Volumetric analysis of GEM enrichment at ER-LD contact sites by cryo-ET. Scatter plot shows the fold enrichment of GEMs at contact sites per tomogram. Bar represents the overall enrichment calculated from summed volumes. Corresponding numerical data are provided in Supplementary Table 2. *n* = 19 tomograms from 7 cells, 5 experiments. **c**, Selection of cells for cryo-ET based on GEM2 fluorescence. Maximum intensity projection image of seipin-sfGFP cells expressing GEM2 on a grid, treated with rapalog for 10 h, oleic acid for 1 h, and imaged by widefield microscopy before freezing. Insets show three cells with varying GEM2 levels, from which cryo-ET data were collected. Right, mean fold enrichment of GEMs at ER-LD contact sites per cell as a function of total GEM fluorescence as imaged before freezing. Each dot represents a cell. Numbers indicate cells highlighted in fluorescence image on the left. **d**, Registration of tomogram with lamella image via surface ice contaminants (arrowheads). On-lamella fluorescence signals are transformed based on the calculated affine transform. **e**, Annotated GEM2 particles compared with registered fluorescence in tomograms of seipin-sfGFP and mEGFP-Ki-67 cells. Poorly colocalising GEM fluorescence could arise from a combination of low objective numerical aperture, optical aberrations, image drift during acquisition, artefacts in Airyscan processing, sample distortion during handling or imaging, and registration errors. Source data

To quantify the specificity of GEM2 labeling, we further assessed whether the presence of GEMs at ER-LD contact sites could be accounted for by random occurrence. Summing over 19 tomograms, 40 of the observed 91 GEM particles localized within 50 nm of a contact site, a volume that comprised only 0.32% of the total imaged cellular volume. We thus estimated a roughly 138-fold enrichment of GEM particles around an ER-LD contact site (Extended Data Fig. 8a and Supplementary Table 2), in agreement with relative labeling index measurements of endogenous seipin localizations via immunogold labeling in room-temperature EM^41–43^, indicating high specificity. In the face of high background from GEMs not bound to the target, such as for the low-abundance seipin, we note that the enrichment of GEMs at contact sites correlated inversely with GEM expression level in cells, as evaluated by widefield fluorescence imaging before freezing (Extended Data Fig. 8b,c). This correlation is corroborated in our light microscopy analyses of GEM recruitment to Ki-67, Nup96 and seipin (Extended Data Fig. 5b,e,h). Therefore, selecting cells based on GEM fluorescence for cryo-ET imaging represents an efficient strategy for fine-tuning labeling specificity, especially for low-abundance protein targets.

### On-lamella CLEM assists targeting specific GEM2 labeling

**Fig. 4:**
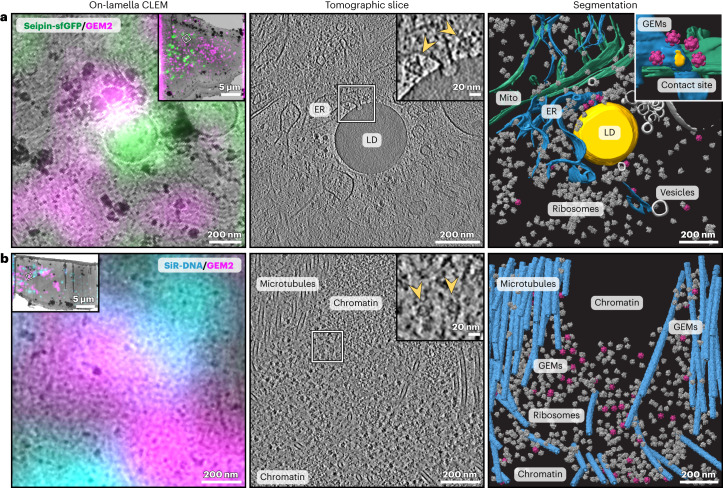
On-lamella CLEM-assisted localization of GEM2 labeling. **a**, Localization of GEM2-labeled seipin. The left shows a TEM image of an FIB lamella superposed with cryo-Airyscan fluorescence, registered via LD signals in the reflected light image of the lamella, in the same view as the tomographic slice and segmentation shown on the right. Colocalizing GEM2 (magenta) and seipin-sfGFP (green) fluorescence signals indicate the location of GEM2-labeled seipin. In the inset, a white box indicates the area of cryo-ET data acquisition. The middle shows a tomographic slice from the indicated area. The right shows the segmentation of mitochondria (green), ER (blue) and LD (yellow). Ribosomes (gray) and GEMs (magenta) are represented by subtomogram averages pasted into the tomogram according to their refined poses. Insets show a closer view of the GEM-decorated ER-LD contact site (arrowheads in middle inset indicate GEMs), and viewed from a different orientation in the right inset. **b**, Localization of GEM2 on the EGFP-Ki-67-coated mitotic chromosome periphery. The left shows the TEM lamella image, superposed with cryo-Airyscan fluorescence, in the same view as the tomographic slice and segmentation shown on the right. In the middle, the inset shows a closer view of two GEM particles (arrowheads) close to the chromatin periphery. The right shows segmentations of microtubules (blue). Ribosomes (gray) and GEMs (magenta) are represented by subtomogram averages pasted into the tomogram.

With 60 Halo-tags per particle, GEMs provide an additional opportunity for cryo-CLEM by contributing strong fluorescence in the roughly 200-nm-thin FIB lamellae to guide cryo-ET data acquisition (Fig. 4). By imaging where GEM2 fluorescence colocalized with seipin-sfGFP signal on lamellae, we visualized multiple GEMs surrounding an ER-LD contact site (Fig. 4a). The arrangement of GEMs observed in this tomogram strongly suggests that the transmembrane homo-oligomeric seipin complex encircles the contact site, as previously hypothesized^38,44^. This provides new insights into the in situ conformation of seipin at the ER-LD contact site, valuable for deriving a potential mechanism of LD biogenesis and homeostasis. Likewise, by imaging where the silicon rhodamine (SiR) DNA dye fluorescence juxtaposed with GEM2 fluorescence in EGFP-Ki-67 cells, we identified regions of the mitotic chromosome periphery labeled with GEMs for cryo-ET imaging (Fig. 4b). Finally, in both examples, by registering between the transmission electron microscopy (TEM) image of the lamella overlaid with the fluorescence data acquired post-FIB milling and the tomograms, we could confirm that most GEM particles annotated in the cryo-ET data coincided with fluorescence (Extended Data Fig. 8d,e). Therefore, GEM fluorescence in lamellae can be harnessed in combination with other fluorescence signals, such as those of the target or associated cellular structures, to pinpoint specific labeling events and to guide targeted cryo-ET data acquisition.

### GEM-tagging of cellular compartment subdomains

**Fig. 5:**
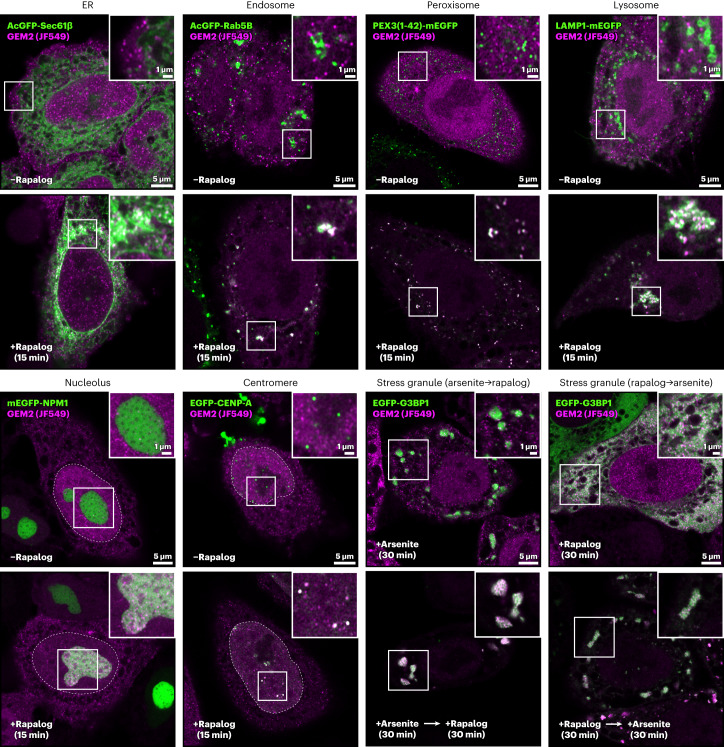
GEM2 labeling of GFP-tagged proteins specific to different compartments in human cells. GFP-tagged proteins were overexpressed in HeLa cells with a GEM2/adaptor AAVS1 knock-in. Cells were cultured for 48 h after transfection of GFP-tagged protein plasmids. Rapalog treatment times are indicated for each target. For G3BP1, GEM2 labeling was induced before or after induction of stress granule formation via arsenite treatment. These are representative images, and the experiment was performed twice with similar results.

**Extended Data Fig. 9:**
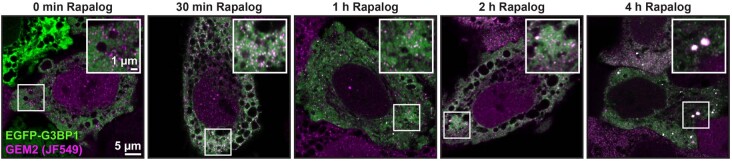
Clustering of cytosolic G3BP1 during prolonged GEM labelling. HeLa cells with GEM2/adaptor AAVS1 knock-in were cultured 48 h after transfection of EGFP-G3BP1 plasmid. GEM2 and adaptor expression from the AAVS1 locus was induced by 24 h doxycycline treatment. Cells were treated with rapalog for the indicated times under non-stress conditions. Formation of GEM2-EGFP-G3BP1 clusters in the cytoplasm are apparent at 4 h. Two experiments were performed with similar results.

As our test cases illustrate, GEMs can localize organelle subdomains such as ER-LD contact sites by labeling endogenous seipin, as well as macromolecular assemblies formed by unstructured or flexible proteins such as Ki-67 on the mitotic chromosome surface. To further assess the general applicability of GEM2, we tested labeling at a variety of cellular substructures, using a stable cell line that inducibly expresses GEM2 and adaptor protein in combination with transient transfection of different GFP-tagged target proteins. The proteins tested localize to membrane subdomains of the ER (Sec61β), endosomes (Rab5), peroxisomes (Pex3) and lysosomes (LAMP1), to nuclear centromeres (CENP-A) or membraneless compartments such as the nucleolus (NPM1), and to cytoplasmic stress granules (G3BP1). Fluorescence imaging showed that GEMs labeled each overexpressed target protein in a ligand-controlled manner (Fig. 5). Notably, while labeling of diffuse G3BP1 in the cytoplasm for 30 min did not visibly affect its partitioning into stress granules on subsequent induction of oxidative stress^3^, and while labeling of G3BP1 was also possible in preassembled stress granules (Fig. 5), excessive rapalog treatment times of 2–4 h resulted in aberrant clustering of the soluble protein pool (Extended Data Fig. 9). These examples illustrate that GEM2 can be applied to localize a wide range of subcellular compartments and subdomains thereof, while careful optimization and assessment of phenotype, which we detailed for Ki-67, Nup96 and seipin can be conducted effectively using light microscopy.

## Discussion

Here we presented a ligand-inducible GEM labeling strategy for intracellular proteins that enables localization precision on the scale of 10–25 nm at organelle subdomains in cryo-ET. Based on a 25-nm icosahedron-forming encapsulin scaffold, the GEM2 tag exhibits fast cytoplasmic diffusion and is readily identifiable in tomograms visually and using a dedicated CNN. We demonstrated that the tag can label endogenous and overexpressed GFP-fusion proteins in the cytoplasm and nucleus of different human cell lines. Thus, alongside current solutions including DNA origami labels^15^, mainly used extracellularly, and ferritin tags^13,14,19^, which require the availability of free iron, the ligand-controlled GEM2 tag provides an orthogonal strategy for labeling intracellular targets under close-to-native conditions.

Given their 25-nm size and multimeric nature, the use of GEM2 tags is not without potential artifacts or limitations. First, while the labeling is ligand-induced and therefore not constitutive, once bound to the target protein, a GEM particle could still hamper diffusion, alter structure or interfere with function within the timeframe of the experiment. With excessive labeling times, clustering of the target due to multivalent GEM binding also becomes likely. Given these concerns, it is important to optimize for each target toward a minimal induction time that preserves phenotype and at the same time provides adequate GEM binding. Assessment of labeling efficiency can be effectively carried out by light microscopy before commencing the cryo-ET experiment. Our analyses based on endogenous Ki-67, Nup96 and seipin suggest that the optimal GEM2 labeling time can vary between minutes and hours, and inversely correlates with target protein abundance.

Second, inherent to all labeling strategies where the tag is introduced separately from the target, unbound tags contribute to nonspecific background and therefore limit specific localization of the target protein. Future solutions may benefit from the incorporation of elements that trigger a change in fluorescence and/or structure of the tag on target binding. While this will require further engineering, we have presented several workarounds using the GEM2 system. We showed with seipin and Ki-67 that GEM fluorescence on lamellae can be leveraged to pinpoint colocalization events for cryo-ET imaging and to support the identification of specifically-bound GEM particles in tomograms. We also showed that the proportion of target-bound GEMs can be increased by selecting cells with low GEM abundances via fluorescence-based cell sorting or imaging on grids before freezing. Beyond these approaches, the specificity of target localization through GEMs can be assessed from the fold enrichment of GEM particles at a defined subcellular location, analogous to the relative labeling index approach as used in immunogold labeling^45^.

Third, while we have successfully deployed the GEM2 tag to label cytosolic subdomains of membranous organelles, and membraneless compartments in the nucleus and cytoplasm, the GEM2 tag is not currently designed to localize to the lumen of membranous organelles.

**Extended Data Fig. 10:**
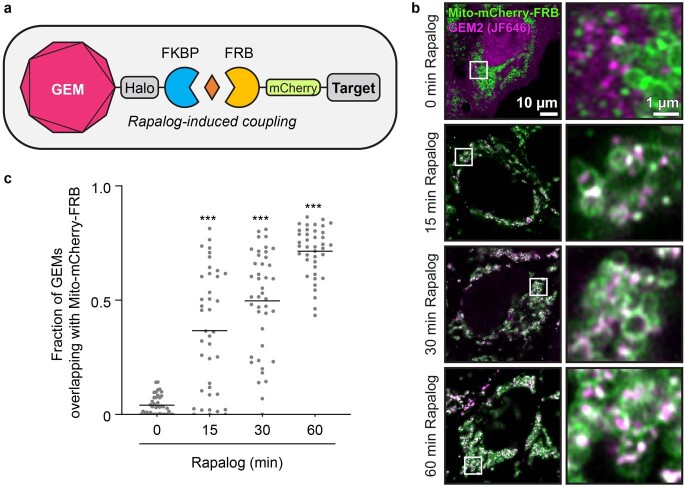
Nanobody-free GEM2 labelling system. **a**, Schematic of the system. **b**, HeLa cells with doxycycline-inducible GEM2-Halo-FKBP and Mito-mCherry-FRB AAVS1 knock-in were treated with rapalog for the indicated times. **c**, Analysis of b. Lines indicate mean, *n* = 41, 40, 42, 40 cells per treatment (left to right), 2 experiments. ***P < 0.0001, Kruskall-Wallis test followed by Dunn’s test, compared to 0 h rapalog treatment. Source data

Finally, the symmetric structure of the encapsulin-derived GEM2 tag and the semiflexible nature of the linkers prevent unambiguous localization of the tagged target protein, rendering the direct use of GEMs for particle picking towards downstream structural analysis by subtomogram averaging currently impossible. However, we envisage the modular nature of the GEM system will enable further customization. For example, nanobody-free labeling can be realized by fusion of FRB directly to the target protein, removing the need for an adaptor protein and providing the additional advantage of a shorter linker, which potentially affords more precise localizations (Extended Data Fig. 10). Further development of a more rigid linker or incorporation of asymmetric elements can potentially aid in subtomogram analysis of structurally defined complexes. With increasing throughput in the generation of pristine cryo-ET data, and availability of robust particle localization algorithms, protein structure prediction and macromolecular structure determination approaches^11,12,46–51^, GEMs set the stage for the development of tags that can be harnessed to corroborate the molecular identity of structures determined de novo inside cells at moderate resolutions, facilitate manual particle picking and ultimately enable the training of dedicated CNNs for detection of the target structure in the absence of GEMs, expanding the potential of cryo-ET to applications where previous structural knowledge is lacking.

## Methods

A step-by-step protocol is available in Supplementary Information.

### Mammalian cell culture

HeLa cell lines were derived from a previously described HeLa Kyoto cell line^52^. HeLa cells endogenously tagged with mEGFP at the N terminus of Ki-67 were previously described^36^. For generating HeLa cells stably expressing EGFP on the outer mitochondrial membrane, cells were transfected with a plasmid harboring an import signal of yeast mitochondrial outer membrane Tom70p fused to EGFP^53^ using pEGFP-N1 as a backbone (Mito-EGFP) using polyethylenimine (PEI) Max (Polysciences) and cultured for 2 days. Cells were selected using 1 mg ml^−1^ G418 (Thermo Fisher Scientific) for 2 weeks and GFP-positive cells were then isolated by fluorescence-activated cell sorting (FACS) using BD FACSAria Fusion, expanded and validated using fluorescence microscopy. HeLa cells were cultured in Dulbecco’s modified medium (DMEM) (Gibco) containing 10% (v/v) fetal bovine serum (FBS) (Thermo Fisher Scientific), 1% (v/v) penicillin–streptomycin (Gibco) and 1 mM sodium pyruvate (Thermo Fisher Scientific). U2OS cells with knock-in of mEGFP into endogenous locus of Nup96 (ref. ^54^) were cultured in McCoy’s 5A modified medium (McCoy; Thermo Fisher Scientific), supplemented with 10% (v/v) FBS, 1% (v/v) penicillin–streptomycin, 1 mM sodium pyruvate and 1% MEM nonessential amino acids (Thermo Fisher Scientific). Sum159 cells endogenously tagged with sfGFP at the N terminus of seipin and seipin knock-out cells have been previously described^55,56^. Sum159 cells were maintained in DMEM/F-12 GlutaMAX (Thermo Fisher Scientific) supplemented with 5 mg ml^−1^ insulin (Cell Applications), 1 mg ml^−1^ hydrocortisone (Sigma), 5% FBS (v/v), 50 mg ml^−1^ streptomycin and 50 U ml^−1^ penicillin. All cells were cultured at 37 °C in a 5% CO_2_-containing atmosphere. These conditions were used for all live cell imaging experiments.

### GEM library screening

Codon-optimized synthetic GEM genes (Biomatik) were subcloned into pcDNA3.1(+) for transient expression under a cytomegalovirus promoter as Halo-FRB (T2098L) fusions in HeLa cells using PEI Max (Supplementary Table 1). All cell lines and plasmids described in this study are listed in Supplementary Tables 3 and 4, respectively. All primers used for cloning are listed in Supplementary Table 5. Expression plasmids for GEM2, GEM4, GEM7, GEM22 and GEM23 are available as Addgene plasmids nos. 197056, 197057, 197058, 197059 and 197060. Two days after transfection, cells were labeled with 100 nM Halo-tetramethylrhodamine (TMR) (Promega) for 20 min and then with 0.2 µg ml^−1^ Hoechst 33342 (Thermo Fisher Scientific) for 30 min before imaging. For screening of ligand-inducible GEM recruitment to GFP, HeLa cells stably expressing Mito-EGFP were transfected with a doxycycline-inducible GEM-Halo-FRB-IRES-adaptor gene cassette, hereafter referred to as GEM–adaptor, encoding the GEM-Halo-FRB fusion, an EMCV-derived IRES sequence^57^, and the adaptor protein (FKBP-SNAP-vhhGFP4) (ref. ^20^) under a TREtight promoter^58^. Cells were treated with 2 μg ml^−1^ doxycycline for 24 h and then labeled with 100 nM Halo-TMR ligand and 100 nM SNAP-Cell 647-SiR ligand (New England Biolabs) for 20 min. To induce GEM recruitment to GFP, cells were treated with 0.5 µM rapalog (TAKARA) for 15 and 30 min. Cells were washed with phosphate buffered saline (PBS), fixed with 3.7% formaldehyde in PBS for 15 min and washed with PBS three times. Single *z*-plane images were acquired on a Zeiss LSM980 confocal microscope, with an Airyscan detector and Plan-Apochromat ×63/1.4 numerical aperture (NA) Oil DIC M27 objective. Images were processed with identical settings in each data set using Zen Blue (Zeiss). Cell boundaries were manually determined in Fiji.

### Plasmid construction for CRISPR–Cas9 genome editing

To generate a GEM2 donor plasmid for knock-in into the human adeno-associated virus integration site 1 (AAVS1), Addgene plasmid no. 129715 (ref. ^59^) was used for Gibson assembly with two gene cassettes: the GEM2–adaptor cassette described above; and rtTA3 transactivator and selection markers separated by 2A self-cleaving peptides (rtTA3-P2A-PuroR-T2A-Thy1.1) under the EF1α promoter. To generate a donor plasmid for knock-in of GEM2-Halo-FKBP in the nanobody-free system, GEM-Halo-FKBP was inserted under the EF1α promoter into Addgene plasmid no. 129715 after initial insertion of an additional multiple cloning site. To generate a donor plasmid for parallel knock-in of Mito-mCherry-FRB into the AAVS1, Mito-mCherry-FRB was inserted under the EF1α promoter into Addgene plasmid no. 129719 after initial insertion of an additional multiple cloning site. Two Cas9–single-guide RNA (sgRNA)-expressing plasmids to be used with the AAVS1 donor plasmids were generated based on Addgene plasmid no. 129725 (ref. ^59^) with sgRNA sequences derived from Addgene plasmid nos. 129726 and 129727 (AAVS1-1 target sequence: ACCCCACAGTGGGGCCACTA GGG and AAVS1-2 target sequence: GTCACCAATCCTGTCCCTAG TGG). Donor plasmids constructed in this study are available as Addgene plasmids nos. 197061–197067.

### Generation of GEM2-expressing HeLa cell lines via CRISPR–Cas9 genome editing

HeLa cells stably expressing Mito-EGFP or endogenously tagged mEGFP-Ki-67 were electroporated with the two Cas9–sgRNA-expressing plasmids, 5 µg each, and 7.5 µg GEM2 donor plasmid using the Neon Transfection System (Thermo Fisher Scientific) with 3 × 10 ms pulses at 1,300 V. Electroporated cells were selected with 0.5 µg ml^−1^ puromycin for 2 weeks. To select for the presence of surface protein Thy1.1, trypsinized cells were incubated with 0.4 µg ml^−1^ anti-Thy1.1 antibody conjugated with allophycocyanin in FACS buffer (2% FBS and 0.5 µM EDTA in PBS) for 30 min on ice. Then, allophycocyanin-positive cells were isolated onto a 96-well plate by FACS. Expanded cells were assessed by light microscopy for cell morphology, GEM2 expression on doxycycline treatment and GEM2-GFP coupling efficiency on rapalog treatment. To generate cell lines for nanobody-free GEM2 labeling, HeLa cells were electroporated with four plasmids, 5 µg each: the same two Cas9–sgRNA-expressing plasmids as above, GEM-Halo-FKBP AAVS1 donor plasmid with a puromycin resistance gene and Mito-mCherry-FRB AAVS1 donor plasmid with a blasticidin resistance gene. To select for parallel knock-ins into different alleles of the AAVS1 locus, cells were treated with 0.5 µg ml^−1^ puromycin and 6 µg ml^−1^ blasticidin (Sigma) for 2 weeks. For FACS, cells were stained with 50 nM Halo-TMR for 30 min. Cells showing strong red fluorescence, likely due to the presence of both mCherry and TMR staining, were isolated onto a 96-well plate. Expanded cells were validated by microscopy for cell morphology, GEM2 and Mito-mCherry expression, and GEM2-Mito-mCherry coupling efficiency on rapalog treatment.

### Generation of GEM2-expressing Sum159 cell lines via CRISPR–Cas9 genome editing

Sum159 seipin-sfGFP cells were transfected with the two Cas9–genomic RNA-expressing plasmids, 0.7 µg each, and 1.05 µg GEM2 donor plasmid as above, using Lipofectamine LTX with Plus reagent. One day later, cells were treated with 1 μg ml^−1^ puromycin for 7 days. Thereafter, a single clone was isolated by dilution cloning and this clone was further FACS-sorted for low level GEM2 fluorescence. For FACS sorting, GEM2 expression was induced by treatment with 0.2 μg ml^−1^ doxycycline for 24 h and GEMs were stained for 1 h with 200 nM Halo-JFX646 (ref. ^60^).

### Transient expression of GEM2 in Nup96 U2OS cells

U2OS cells endogenously expressing Nup96-mEGFP were transfected with the GEM2 donor plasmid using PEI and cultured for 1 day, followed by treatment with 2 µg ml^−1^ doxycycline for 24 h.

### GEM labeling of overexpressed GFP-tagged proteins in different cellular regions

For overexpression of mEGFP-NPM1, the coding sequence of NPM1, derived from pDONR223-NPM1 (a gift from D.W. Gerlich), was inserted after mEGFP into a pFUGW vector by Gibson assembly. For overexpression of EGFP-G3BP1, the coding sequence of G3BP1, derived from pcDNA3.1-mCherry-G3BP1 (ref. ^61^) (a gift from A.A. Hyman), was inserted after EGFP in a pIRESpuro2 vector by restriction cloning. GEM2 knock-in cells were transfected with mEGFP-NPM1, EGFP-G3BP1, EGFP-CENP-A (a gift from K.F. Sullivan), AcGFP-Sec61β (Addgene plasmid no. 15108), AcGFP-Rab5B (no. 61802) (ref. ^62^), LAMP1-mEGFP (no. 120172), and PEX3(1-42)-mEGFP (no. 120174) (ref. ^63^), using PEI and cultured for 1 day, followed by treatment with 2 µg ml^−1^ doxycycline for 24 h. GEMs were labeled with 50 nM Halo-JF549 (Promega) for 1 h. To induce GEM recruitment to GFP, cells were treated with 0.5 µM rapalog for the indicated times (Fig. 5) and fixed with 3.7% formaldehyde in PBS for 15 min. After washing with PBS three times, DNA was stained with 0.2 µg ml^−1^ Hoechst 33342 for 15 min at room temperature. Images were acquired on a Zeiss LSM980 confocal microscope with an Airyscan detector and Plan-Apochromat ×63/1.4 NA Oil DIC M27 objective.

### Fluorescence recovery after photobleaching analysis of GEM2 mobility

GEM2 knock-in cells stably expressing Mito-EGFP were stained with 100 nM Halo-TMR for 30 min after treatment with 2 µg ml^−1^ doxycycline for 1 day. Photobleaching was carried out on a Zeiss LSM780 using a Plan-Apochromat ×63/1.4 NA Oil DIC M27 oil-immersion objective. Bleaching was performed in a 4 µm circular region after capturing initial ten frames using a laser intensity 160-fold higher than the laser intensity used for image acquisition. Images were acquired every 50 ms for the course of the experiment. Intensities were normalized according to a background region outside the cell as described by Halavatyi and Terjung^64^. A single exponential recovery curve was fitted to the mean of single-normalized intensities over time using FRAPAnalyser (https://github.com/ssgpers/FRAPAnalyser).

### Kinetics assays of GEM recruitment to GFP-tagged proteins

To assess the kinetics of GEM recruitment to Mito-EGFP, cells were seeded onto LabTek eight-well plates (Thermo Fisher Scientific) and treated with 2 µg ml^−1^ doxycycline for 2 days. GEM-Halo-FRB was labeled with Halo-TMR and then treated with 0.5 µM rapalog for 15, 30 and 60 min. Cells were washed with PBS, then fixed with 3.7% formaldehyde in PBS for 15 min and washed with PBS three times.

To assess the kinetics of GEM2 recruitment to Ki-67 on mitotic chromosomes, cells were seeded onto poly-l-lysine-coated (Sigma) LabTek eight-well plates and treated with 2 mM thymidine (Sigma) and 2 µg ml^−1^ doxycycline for 24 h. Cells were washed with prewarmed medium three times and cultured in fresh medium supplemented with 2 µg ml^−1^ doxycycline and 10 µM S-trityl-l-cysteine (Sigma) for 14–16 h to enrich for mitotic cells^65^. Cells were labeled with Halo-TMR and then treated with 0.5 µM rapalog for 15, 30 and 60 min. Cells were washed with PBS, then fixed with 3.7% formaldehyde in PBS for 15 min and washed with PBS three times.

To assess the kinetics of GEM2 recruitment to Nup96, transfected cells were stained with Halo-JF646 (Promega) and treated with 0.5 µM rapalog for 1, 6 and 12 h. Cells were washed with PBS, fixed with 3.7% formaldehyde in PBS for 15 min and washed with PBS three times.

To assess the kinetics of GEM2 recruitment to seipin, cells were seeded onto ibidi eight-well plates and treated with 0.2 µg ml^−1^ doxycycline for 24 h. Cells were then treated with 0.5 µM rapalog for 1, 5 and 12 h, 500 µM oleic acid for 1 h and stained with 200 nM Halo-JFX646 for 1 h. Cells were washed with PBS, then fixed with 4% formaldehyde in PBS for 15 min, washed with PBS three times and quenched with 50 mM NHCl_4_. Cells were kept in PBS until imaging.

Images were acquired on a Zeiss LSM980 confocal microscope, equipped with an Airyscan detector and Plan-Apochromat ×63/1.4 NA Oil DIC M27 objective. For Mito, Ki-67 and Nup96, single confocal *z*-planes were acquired and analyzed. For seipin, *z*-stacks covering the whole cell were acquired and analyzed. Images were processed with identical settings in each data set using Zen Blue software (Zeiss). Cell boundaries were manually determined in Fiji. GEMs and target proteins (Mito, Ki-67, Nup96 or seipin) were segmented using ilastik^66^ and the fraction of overlapping areas was analyzed using CellProfiler^67^.

### Mitotic chromosome area assay

To assess whether GEM recruitment to Ki-67 affects chromosome dispersion, cells were seeded onto LabTek eight-well plates and treated with 2 µg ml^−1^ doxycycline for 2 days. GEMs and DNA were stained with Halo-TMR and SiR-DNA (Spirochrome), respectively. Then, cells were arrested in mitosis with 200 ng ml^−1^ nocodazole for 2 h, and subsequently treated with 0.5 µM rapalog for 15, 30 and 60 min. Z-stacks of whole live cells were acquired with 3 µm steps on Zeiss LSM780 using an EC Plan-Neofluar ×40/1.30 NA Oil DIC M27 oil-immersion objective. To deplete Ki-67, mEGFP-Ki-67 knock-in cells were transfected with small interfering RNA (siRNA) against Ki-67 (ref. ^36^) and cultured for 2 days. Cells were arrested in mitosis with 200 ng ml^−1^ nocodazole for 2 h and then imaged with the same settings on a Zeiss LSM780 using an EC Plan-Neofluar ×40/1.30 NA Oil DIC M27 oil-immersion objective. Mitotic cells were manually cropped and the center slice was selected based on the mean intensity of the DNA signal. Mitotic chromosomes were segmented and their ensemble area in pixels was analyzed using ilastik^66^. For Ki-67 knockdown, cells were transfected with previously described siRNAs (sense sequence: CGUCGUGUCUCAAGAUCUAtt, Thermo Fisher Scientific Silencer Select, siRNA ID s8796) ^36^ using Lipofectamine RNAiMax (Invitrogen) and incubated for 48 h. XWneg9 (sense sequence: UACGACCGGUCUAUCGUAGtt, Thermo Fisher Scientific Silencer Select, custom synthesis) was used as a nontargeting siRNA control at a final concentration of 10 nM. Ki-67 siRNA was used at final concentrations of 10, 1 and 0.1 nM. For analysis of mitotic chromosome area, cells were treated with 200 ng ml^−1^ nocodazole for 2 h and then imaged and analyzed as above.

### Importin β binding domain import assay

To assess whether GEM recruitment to Nup96 affects nuclear transport, U2OS cells were seeded onto LabTek eight-well plates, transfected with importin β binding domain (IBB)-mCherry and the GEM2 donor plasmid, and then cultured for 1 day followed by treatment with 1 µg ml^−1^ doxycycline for 1 day. Cells were then treated with 0.5 µM rapalog for 1, 6 and 12 h and GEMs were stained with 100 nM of Halo-JF646 (Promega) for 1 h before fixation. Fixed cells were washed with PBS, fixed with 3.7% formaldehyde in PBS for 15 min and then washed with PBS three times. DNA was stained with 0.2 µg ml^−1^ Hoechst 33342 for 15 min at room temperature. Images were acquired by a Zeiss LSM980 confocal microscope with an Airyscan detector and Plan-Apochromat ×63/1.4 NA Oil DIC M27 objective. Cell boundaries and nuclei were determined manually and by Otsu thresholding in Fiji^68^, respectively. Cytoplasmic segmentations were defined by subtracting the nucleus segmentations from whole cell segmentations. Relative mean IBB intensities were calculated as mean IBB intensity in the nucleus divided by the mean IBB intensity in the cytoplasm.

### LD size quantification

To assess whether GEM recruitment to seipin affects LD sizes, cells were seeded onto ibidi eight-well dishes, and treated with 0.2 µg ml^−1^ doxycycline for 24 h and 0.5 µM rapalog for the indicated times to induce seipin-GEM tethering. Cells were also treated for 1 h with 500 µM oleic acid to induce LD biogenesis and simultaneously stained with 200 nM Halo-JFX646. Cells were then washed with PBS twice, fixed in 4% PFA in PBS for 20 min and washed again with PBS twice. Nuclei were stained with Hoechst for 5 min at room temperature and LDs with 0.2 µg ml^−1^ LD540 (ref. ^69^) in PBS for 20 min at room temperature. *Z*-stacks of whole cells were acquired with 0.3 µm steps on a Nikon Ti-E widefield microscope with CFI P-Apo DM ×60/1.4 NA Lambda oil objective. LDs were segmented with Ilastik, LD sizes per cell were analyzed using CellProfiler and Object Analyser as described^38^.

### FCS-calibrated imaging

To quantify GFP-tagged protein abundances in living cells, Mito-EGFP, mEGFP-Ki-67, Nup96-mEGFP and seipin-sfGFP cells were seeded into individual chambers of an 18-well ibidi glass bottom slide in the respective media alongside nontransfected HeLa wild-type cells for estimation of background photon counts, and HeLa wild-type cells transfected with mEGFP for calibration. Before imaging, medium was changed to HEPES-based imaging medium (30 mM HEPES pH 7.4, Minimum Essential Eagle medium (Sigma), 10% (v/v) FBS, 1× Minimum Essential Medium nonessential amino acids (Gibco)) containing 100 nM 5-SiR-Hoechst^70^ (gift from G. Lukinavičius). For Mito-EGFP and Nup96-mEGFP cells, 200 nM 5-SiR-Hoechst and 1 µM Verapamil (Spirochrome) were used. After 1 h, the imaging medium was supplemented with 500-kDa dextran (Thermo Fisher Scientific) conjugated in-house with Dy481XL (Dyomics) to label the extracellular space.

FCS-calibrated imaging was carried out as previously described^39^ on a Zeiss LSM880 using a C-Apochromat ×40 1.20 W Korr FCS M27 water-immersion objective. Confocal volume estimation was carried out by ten 1-min FCS measurements of 10 nM Atto488 (ATTO-TEC) in water. Background fluorescence and background photon counts were determined by FCS measurements in the nucleus and cytoplasm of nontransfected cells. An experiment-specific calibration line was generated by repeated nucleus and cytoplasm-targeted FCS measurements of wild-type cells expressing a range of levels of mEGFP. This allowed for the determination of an experiment-specific internal calibration factor with which measured GFP fluorescence intensities could be converted into protein concentrations.

Z-stacks of whole cells were acquired in the GFP, 5-SiR-Hoechst, Dy481XL and transmission channels. A previously established 3D cell segmentation pipeline^71^ using software FCSRunner, MyPic, Fluctuation Analyzer 4G, FCSFitM, FCSImageBrowser and FCSCalibration was adapted to segment individual cells in large fields of view and to extract GFP fluorescence intensities for conversion into absolute protein numbers (Brunner et al., unpublished).

### Cryo-ET sample preparation

For all experiments, Au SiO_2_ R1.2/20 Quantifoil grids, 200 mesh, were micropatterned with 30-µm fibronectin circles in the center of grid squares, as described in ref. ^72^.

For Mito-EGFP, cells seeded in a 6 cm dish were treated with 2 µg ml^−1^ doxycycline for 1 day. Then, 2.0 × 10^5^ trypsinized cells were seeded onto grids in ibidi 35 mm low dishes (six grids per dish) and incubated in 1 ml of medium for 1 h. After cell attachment, grids were transferred to new ibidi 35 mm low dishes and further cultured in 1 ml of medium containing 2 µg ml^−1^ doxycycline for 1 day. Cells were stained with 50 nM Halo-JF646 for 1 h to label GEMs and then treated with 0.5 µM rapalog for 30 min. To verify GEM2 expression and labeling before freezing, fluorescence imaging was performed on a Zeiss Axio Observer microscope with a Plan-Apochromat ×63/1.4 NA oil objective. Cells were frozen within 30–60 min after rapalog treatment.

For mEGFP-Ki-67, cells were synchronized in a 6 cm dish before seeding by double thymidine block at the G1/S boundary: cells were treated with 2 mM thymidine for 24 h, released and cultured in fresh medium for 8 h and treated again with 2 mM thymidine for 16–24 h. For seeding, 2.0 × 10^5^ trypsinized cells were seeded onto grids in ibidi 35 mm low dishes (six grids per dish) and incubated in 1 ml of fresh nonarresting medium for 1 h. The grids were transferred to 1 ml of fresh nonarresting medium in ibidi 35 mm low dishes after cell attachment. Four hours postrelease, cells were stained with Halo-TMR for 20 min and then DNA was stained with 0.2 μM SiR-DNA until freezing. To monitor progress into mitosis, fluorescence montages of grids were recorded on a LSM780 confocal microscope with an EC Plan-Neofluar ×20/0.50 NA objective at 37 °C under 5% CO_2_ from 8.5 h postrelease. Cells were frozen within 5–10 min of observing mitotic entry, typically 9–9.5 h postrelease (Fig. 4b and Extended Data Fig. 4b). In a subset of experiments (Fig. 2b), 200 nM nocodazole was added to arrest cells in mitosis alongside SiR-DNA treatment.

For Nup96-mEGFP, cells transfected with the GEM2 donor plasmid in a 6 cm dish were treated with 2 µg ml^−1^ doxycycline for 1 day. Then, 2.0 × 10^5^ trypsinized cells were seeded onto grids in ibidi 35 mm low dishes (six grids per dish) and incubated in 1 ml of medium for 1 h. Grids were transferred to 1 ml of fresh medium in ibidi 35 mm low dishes after cell attachment and treated with 0.5 µM rapalog for 6 h. Fluorescence imaging was carried out on a LSM780 confocal microscope with a C-Apochromat ×63/1.20 W Corr M27 at 37 °C under 5% CO. Cells were frozen 7–9 h after rapalog treatment.

For seipin-sfGFP, cells in 6 cm dishes or 25 cm^2^ culture flasks were treated with 0.2 μg ml^−1^ doxycycline for 12–16 h. Then, 4.0 × 10^5^ trypsinized cells were seeded onto grids in ibidi 35 mm low dishes (4–5 grids per dish) and incubated in 1 ml of medium for 20–30 min. After cell attachment, grids were transferred to a new ibidi 35 mm dish and treated with 0.5 µM rapalog and 0.2 μg ml^−1^ doxycycline for 10 h. Cells were additionally treated with 200 nM Halo-JFX646 for 1.5–2 h and oleic acid for 45–60 min before freezing. During Halo-JFX646 labeling, montages of the grids were acquired for GEM2 fluorescence on a Zeiss Axio Observer microscope with a Plan-Apochromat ×63/1.4 NA oil objective, or a Nikon Ti-E widefield microscope with a CFI P-Apo ×40/0.95 NA air objective, at 37 °C under 5% CO. For comparison of GEM2 fluorescence between grids (Extended Data Fig. 8c), intensities per cell were normalized against the mean intensity of all cells of the same grid. Initial experiments were performed using a single cell clone. The population was later sorted for 20–60% of the maximum fluorescence. A correction factor was applied accordingly to enable comparison between experiments.

For freezing, in all experiments, 3 µl of medium was added to the cell side of grids before blotting to reduce cell flattening. Grids were blotted from the back for 1–3 s at 37 °C, 90% humidity and plunge-frozen into liquid ethane at −185 °C on a Leica EM GP2 system, clipped into cryo-FIB auto-grids and stored in sealed boxes in liquid nitrogen.

### Cryo-FIB lamella preparation

Cryo-FIB lamellae were prepared using a 45°-pretilt shuttle in an Aquilos FIB-SEM microscope (Thermo Fisher Scientific) as described using SerialFIB^8^. Before milling, metallic platinum was deposited by sputter coating (1 kV, 10 mA, 10 Pa, 15–20 s), and organometallic platinum via the gas injection system at a working distance of 10.6 mm and injection times of 8–11 s. Cells were milled to 1-μm thickness at a stage tilt of 20° with decreasing ion beam currents (1, 0.5 and 0.3 nA, 30 keV) and then thinned all together to a target thickness of 200–250 nm at 50 and 30 pA. Lamellae were thinned at the back at a stage tilt of 21–22°, and finally sputter-coated with platinum (1 kV, 10 mA, 10 Pa, 5–15 s) to reduce charging and beam-induced motion during TEM imaging. Milling progress was assessed by scanning electron microscopy (10 keV, 50 pA).

### Cryo-Airyscan imaging of FIB lamellae

For cryogenic on-lamella CLEM, milled grids were loaded onto a Zeiss LSM 900 Airyscan2 microscope equipped with a Linkam cryo-stage. Using a ×5 air objective in widefield mode, an overview image was acquired to localize lamellae. Next, *z*-stacks with 0.5 µm spacing covering 4–6 µm were acquired in Airyscan mode with 488 and 640 laser lines and a Zeiss Plan-Neofluar ×100/0.75 NA air objective, using a pixel size of 79 nm. In addition, reflection mode images were acquired for each z-plane. For each image, four averages between frames were acquired to increase signal-to-noise ratio. *Z*-stacks were 2D Airyscan processed and maximum intensity projections were generated. Subsequent correlation of Airyscan images and low magnification TEM images (lamella maps) was performed with Icy eC-CLEM^73^ and Fiji BigWarp^74^ using the lamellae shape and features (such as LDs) visible in the reflection images as landmarks.

### Cryo-ET image acquisition

Cryo-TEM montages and tilt series were collected on a Titan Krios G3 (Thermo Fisher Scientific) equipped with a Gatan K2 Summit detector and Quantum energy filter, or a Gatan K3 detector and BioQuantum energy filter, using SerialEM^75^. Grids were loaded such that the lamella pretilt axis aligns with the microscope stage tilt axis. Images were acquired at a pixel size of 3.370 or 3.425 Å/pixel at 1.5–4.0 µm defocus, with an electron dose of 2.0–2.5 e^−^/Å^2^ per image fractionated over 8–10 frames. A dose-symmetric tilt scheme^76^ was used with 2° increments starting from the lamella pretilt (±13°) and an effective tilt range of +56° to −56° using SerialEM^75^. Data were collected with a 70-µm objective aperture or a Volta phase plate, and 20 eV slit width.

### Cryo-ET data processing

CTF estimation and motion correction were performed in WARP^77^. Dose-weighted motion-corrected images were exported for tomographic reconstruction in IMOD^78^ or AreTomo^79^. To train a DeePiCt neural network^12^ for the detection of GEMs, tomograms were binned to a pixel size of 13.48 or 13.70 Å and filtered with the following parameters in EMAN2 (ref. ^80^): filter.low-pass.gauss:cutoff_abs=0.25, filter.highpass.gauss:cutoff_pixels=5, normalize, threshold.clampminmax.nsigma:nsigma=3. Spherical labels of 137 Å radius were generated based on the coordinates of 161 manually picked particles in EMAN2. A DeePiCt network of depth 2, with 32 initial features and batch normalization, was trained with an increasing number of particles and iterative refinement of coordinates via subtomogram averaging (described below). The initial training set contained 161 particles from 39 tomograms, whereas the final training set contained 1284 particles from 71 tomograms. The number of grids and tomograms contributing to this final data set was as follows: Mito, six grids, 12 lamellae and 17 tomograms; Ki-67, three grids, three lamellae and nine tomograms; Nup96, two grids, six lamellae and 20 tomograms; seipin, five grids, nine lamellae and 24 tomograms. Application of the CNN on 17 tomograms of the Mito-EGFP data set yielded 68 peaks after stringent postprocessing, which included thresholding at a value of 0.5, application of a lamella mask and a size filter for connected components of 5,000–50,000 pixels at 13.7 Å/pixel. Of the 68 peaks, two were discarded due to being in a lysosome or mitochondrion, three discarded due to beam-induced damage of the lamella surface and three were cytosolic but did not resemble a GEM visually. On visual inspection of smaller-sized peaks, 63 particles were added manually for subtomogram averaging. Similarly, application of the CNN on 19 tomograms of the seipin-sfGFP data set yielded 108 peaks, of which 26 were discarded: 11 were in lysosomes, or surface ice or damaged particles, and 15 did not resemble a GEM clearly enough. On visual inspection of smaller-sized peaks, nine particles were added manually. For subtomogram averaging, subtomograms at 6.85 Å/pixel with a box size of 128 pixels and 3D CTF models were reconstructed in WARP. Averaging was performed in RELION^81^ with 1,284 particles, I1 symmetry, using a 60-Å-low-pass-filtered map of the encapsulin scaffold (Protein Data Bank (PDB) 6X8M) as a reference. Membranes were segmented by tensor voting with TomoSegMemTV^82^ with manual curation in Amira (Thermo Fisher Scientific). ER-LD contact sites were segmented manually in Amira, defined as the neck-like region where the cytosolic leaflet of the ER bilayer meets the LD monolayer^38^. The distance between a GEM and its target was estimated based on its refined coordinate and distance to the closest annotated membrane or contact-site pixel in Python, subtracting the radius of a GEM particle (12.5 nm). Ribosomes and microtubules were detected with DeePiCt using available models and subtomogram averages obtained after 3D classification and tracing of filaments in MATLAB as described, respectively^12^. For volumetric enrichment analysis of GEMs at ER-LD contact sites, the segmented contact site was dilated by 50 nm in all directions and then masked with a lamella mask, defined geometrically based on the front and back of the lamella visible in the tomogram. Tomograms and segmentations were visualized using IMOD^78^ and ChimeraX^83^.

### Phylogenetic analysis

Phylogenetic analysis of encapsulin amino acid sequences was performed using MAFFT^84^, BMGE^85^, SMS^86^ and PhyML^87^ as in ref. ^16^ and visualized using iTol^88^.

### Statistical analyses

Statistical analyses were performed in GraphPad Prism. All Dunn’s tests performed were two-sided and multiplicity-adjusted for multiple comparisons: all time points were compared against the zero timepoint. Data were tabulated using Microsoft Excel and plotted with GraphPad Prism, RStudio and Gnuplot.

### Reporting summary

Further information on research design is available in the Nature Portfolio Reporting Summary linked to this article.

## Online content

Any methods, additional references, Nature Portfolio reporting summaries, source data, extended data, supplementary information, acknowledgements, peer review information; details of author contributions and competing interests; and statements of data and code availability are available at 10.1038/s41592-023-02053-0.

### Supplementary information

Supplementary Information Supplementary Tables 1–5 and Protocol.

Reporting Summary

Supplementary Video 1 Time-lapse imaging of GEM2 recruitment to Mito-EGFP in HeLa cells. Cells stably expressing Mito-EGFP and GEM2/Adaptor human AAVS1 knock-in were imaged after 48 h of doxycycline induction. Imaging was performed at 1 min intervals upon rapamycin treatment on an Olympus IXplore SpinSR spinning disk confocal microscope using a ×100, 1.35 NA silicone immersion objective. Magenta, GEM2 labeled with Halo-TMR. Green, Mito-EGFP.

Supplementary Video 2 Time-lapse imaging of GEM2 in HeLa cells. GEM2 was transiently expressed in HeLa cells and labeled with Halo-TMR. Imaging was performed at 115 ms intervals on an Olympus IXplore SpinSR spinning disk confocal microscope using a ×100, 1.35 NA silicone immersion objective.

Supplementary Video 3 Cellular cryo-electron tomogram and 3D rendering of GEM2-labeled Mito-EGFP. Tomogram corresponding to slice shown in Fig 3a. Mitochondrial membrane segmentations, labeled Mito, are displayed in green. GEMs are annotated based on refined coordinates from subtomogram averaging in magenta. MT, microtubule. Scale bar, 100 nm.

### Source data

Source data for all plots presented in figures Single file containing all quantitative source data with tabs for each figure item, including main Figs. 1–3 and Extended Data Figs. 3, 5, 6, 8 and 10.

## Data Availability

The subtomogram average of GEM2 is available on the Electron Microscopy Data Bank (EMDB) under entry EMD-16303. Cryo-ET data for GEM labeling of Mito-EGFP, including raw data, tilt series, reconstructed tomograms and GEM coordinates, are deposited on the Electron Microscopy Public Image Archive under entry EMPIAR-11561. A representative tomogram is deposited on EMDB under entry EMD-18194. The atomic model of the *S. elongatus* encapsulin scaffold was obtained from the PDB (6X8M). CRISPR knock-in donor plasmids and GEM2, GEM4, GEM7, GEM22 and GEM23 expression plasmids generated in this study and their full plasmid sequences are available on Addgene under entries nos. 197056–197067. Cell lines generated in this study are available upon request. Source data are provided with this paper.

## Code availability

The DeePiCt CNN model for automated GEM detection and Python script for GEM-target distance analyses are available on GitHub (https://github.com/hermankhfung/GEM).
