# Supplementary material for: Genetically encoded multimeric tags for subcellular protein localization in cryo-EM
Source: Nat Methods. 2023 Nov 6;20(12):1900–8. doi: 10.1038/s41592-023-02053-0 (PMC10703698; doi:10.1038/s41592-023-02053-0)
Supplement: Supplementary file 1 — Supplementary Tables 1–5 and Protocol. [file 41592_2023_2053_MOESM1_ESM.pdf]

nature methods

Article

https://doi.org/10.1038/s41592-023-02053-0

Genetically encoded multimeric tags for subcellular protein localization in cryo-EM

In the format provided by the authors and unedited

Supplementary Table 1: GEM library information. Unless otherwise indicated, all constructs were expressed as GEM-Halo-FRB fusions.

ID	Source organism	Uniprot/RefSeq ID	References
GEM1	*Thermotoga maritima*	Q9WZP2	[23]
GEM2	*Synechococcus elongatus*	Q55032	[24]
GEM3	*Brevibacterium aurantiacum*	A0A1D7VZ58	[25]
GEM4	*Paraburkholderia xenovorans*	I2IC15	[26]
GEM5	*Enterobacteriaceae*	WP_044069342.1	[26]
GEM7	*Rhodospirillum rubrum*	Q2RVS0	[27]
GEM8	*Rhodococcus erythropolis*	A0A0E4ACW8	[28]
GEM9	*Rhodococcus jostii*	Q0SE23	[29]
GEM15	Synthetic (PCquad)	-	[32]
GEM16	Synthetic (Ico532-1) †	-	[33]
GEM17	*Candidatus Scalindua rubra*	A0A1E3XBX5	[30]
GEM22	*Mycobacterium hassiacum*	K5BEG2	[31]
GEM23	Synthetic (I3-01) ‡	-	[34]

† Expressed as an FRB-Halo-GEM fusion

‡ Expressed as FRB-Halo-GEM and FRB-GEM-Halo fusions

Supplementary Table 2: Volumetric analysis of GEM enrichment within 50 nm of ER-LD contact sites in cryo-electron tomograms. Imaged cellular volumes were determined from lamella masks defined geometrically based on cross-sections at the front and back of the lamella per tomogram. ER-LD contact sites, defined as the neck-like region where the cytosolic leaflet of the ER bilayer meets the LD monolayer, were dilated by 50 nm in all directions and masked with the lamella mask for volume calculation.

Tomogram number	Imaged cellular volume (nm^3)	Dilated contact site volume (nm^3)	Contact site volume fraction	Total no. GEMs in tomogram	No. of expected GEMs within 50 nm of contact site	No. of observed GEMs within 50 nm of contact site	Fold enrichment
1	2.54E+08	9E+05	0.0035	7	0.025	3	121.2
2	3.98E+08	2E+06	0.0039	7	0.027	4	147.9
3	2.50E+08	6E+05	0.0024	3	0.007	1	139.9
4	4.70E+08	2E+06	0.0036	3	0.011	3	279.8
5	3.48E+08	2E+06	0.0043	6	0.022	3	116.0
6	2.49E+08	8E+05	0.0033	6	0.020	3	149.4
7	3.31E+08	7E+05	0.0020	3	0.006	1	167.1
8	2.51E+08	8E+05	0.0033	1	0.003	1	302.1
9	2.18E+08	7E+05	0.0031	3	0.006	1	105.9
10	2.31E+08	2E+06	0.0067	3	0.020	1	49.5
11	2.06E+08	9E+05	0.0044	1	0.004	1	225.8
12	2.9E+08	9E+05	0.0030	1	0.003	1	335.5
13	1.61E+08	6E+05	0.0035	3	0.010	2	192.8
14	3.65E+08	9E+05	0.0024	11	0.026	4	151.4
15	2.38E+08	8E+05	0.0034	12	0.040	3	74.4
16	2.91E+08	8E+05	0.0029	2	0.006	2	345.8
17	4.44E+08	9E+05	0.0020	3	0.006	1	164.2
18	3.94E+08	8E+05	0.0021	7	0.015	2	136.8
19	3.51E+08	1E+06	0.0028	9	0.022	3	120.9
**Summary**	5.74E+09†	1.8E+07†	0.0032‡	91†	0.290‡	40†	137.7‡

† Sum total across all tomograms.
‡ Calculated from sum totals.

Supplementary Table 3: Cell lines described in this study.

Cell line name	Comments	Reference	Used in Figs.	Lab ID
HeLa, wild type	Wild-type cells	Originally from S. Narumiya (Kyoto University, Japan)	Extended Data Fig. 2	1
HeLa, Ki-67 KO	Ki-67 knock-out	Published in [36]	Extended Data Fig. 6a,b,d,e	63
HeLa, Mito-EGFP	HeLa stably expressing Mito-EGFP	Generated in this study	Extended Data Fig. 3a–f	423
HeLa, GEM2-Halo-FRB-IRES-adaptor	Knock-in GEM2-Halo-FRB-IRES-adaptor into AAVS1 locus	Generated in this study	Fig. 5, Extended Data Fig. 9	481
HeLa, Mito-EGFP, GEM2-Halo-FRB-IRES-adaptor	HeLa stably expressing Mito-EGFP, Knock-in GEM2-Halo-FRB-IRES-adaptor into AAVS1 locus	Generated in this study	Fig. 1b–d, Fig. 2m–o, Fig. 3a–c, Extended Data Fig. 3g,h, 4a, 7	526
HeLa, mEGFP-Ki-67, GEM2-Halo-FRB-IRES-adaptor	mEGFP-Ki-67 cells published in [36], knock-in GEM2-Halo-FRB-IRES-adaptor into AAVS1 locus	Generated in this study	Fig. 2a–d,m–o, Fig. 4b, Extended Data Fig. 4b, 5a–c, 6a–c, 8d,e	522
HeLa, Mito-mCherry-FRB, GEM2-Halo-FKBP	Knock-in GEM2-Halo-FKBP and Mito-mCherry-FRB into human AAVS1 locus	Generated in this study	Extended Data Fig. 10	539
U2OS, Nup96-mEGFP	Endogenously homozygously labelled Nup96 with C-terminal mEGFP	Published in [54]	Fig. 2e-h, m–o, Extended Data Fig. 4c, 5d–e, 6f–h	466
Sum159, Seipin-sfGFP, GEM2-Halo-FRB-IRES-adaptor	Seipin-sfGFP cells published in [55], knock-in GEM2-Halo-FRB-IRES-adaptor into AAVS1 locus	Generated in this study	Fig. 2i–l, m–o, Fig. 3a–c, Fig. 4a, Extended Data Fig. 4d, 5g–i, 6i–k, 8a–c	hjm105
Sum159, Seipin KO	Seipin knock-out	Published in [56]	Extended Data Fig. 6i, j	hjm106

Supplementary Table 4: Plasmids described in this study.

Name	Comments	Reference	Used in Figs	Lab ID
Mito-EGFP	Based on pEGFP-N1 backbone, used for stable cell line generation	Generated in this study	Fig. 1b–d, Fig. 2m–o, Fig. 3a–c, Extended Data Fig. 3, 4a, 7	840
GEM2-Halo-FRB-IRES-adaptor	Donor vector for human AAVS1 locus, used for knock-in and transient expression	Generated in this study, available as Addgene #197061	Fig. 1–5, Extended Data Fig. 2, 3a,f,g,h, 4, 5, 6, 7, 8, 9	844
GEM4-Halo-FRB-IRES-adaptor	Donor vector for human AAVS1 locus, used for knock-in	Generated in this study, available as Addgene #197062	Extended Data Fig. 3b,f	845
GEM7-Halo-FRB-IRES-adaptor	Donor vector for human AAVS1 locus, used for transient expression	Generated in this study, available as Addgene #197063	Extended Data Fig. 3c,f	862
GEM22-Halo-FRB-IRES-adaptor	Donor vector for human AAVS1 locus, used for transient expression	Generated in this study, available as Addgene #197064	Extended Data Fig. 3d,f	863
FRB-GEM23-Halo-IRES-adaptor	Donor vector for human AAVS1 locus, used for transient expression	Generated in this study, available as Addgene #197065	Extended Data Fig. 3e,f	864
Mito-mCherry-FRB	Donor vector for human AAVS1 locus, used for knock-in	Generated in this study, available as Addgene #197067	Extended Data Fig. 10	943
GEM2-Halo-FKBP	Donor vector for human AAVS1 locus, used for knock-in	Generated in this study, available as Addgene #197066	Extended Data Fig. 10	988
GEM1-Halo-FRB	Based on pcDNA3.1(+) backbone, used for transient expression	Generated in this study	Extended Data Fig. 2	1021
GEM2-Halo-FRB	Based on pcDNA3.1(+) backbone, used for transient expression	Generated in this study, available as Addgene #197056	Extended Data Fig. 2	1022
GEM3-Halo-FRB	Based on pcDNA3.1(+) backbone, used for transient expression	Generated in this study	Extended Data Fig.2	1023
GEM4-Halo-FRB	Based on pcDNA3.1(+) backbone, used for transient expression	Generated in this study, available as Addgene #197057	Extended Data Fig. 2	1024
GEM5-Halo-FRB	Based on pcDNA3.1(+) backbone, used for transient expression	Generated in this study	Extended Data Fig. 2	1025
GEM7-Halo-FRB	Based on pcDNA3.1(+) backbone, used for transient expression	Generated in this study, available as Addgene #197058	Extended Data Fig. 2	1026
GEM8-Halo-FRB	Based on pcDNA3.1(+) backbone, used for transient expression	Generated in this study	Extended Data Fig. 2	1027
GEM9-Halo-FRB	Based on pcDNA3.1(+) backbone, used for transient expression	Generated in this study	Extended Data Fig. 2	1028

GEM15-Halo-FRB	Based on pcDNA3.1(+) backbone, used for transient expression	Generated in this study	Extended Data Fig. 2	1034
FRB-Halo-GEM16	Based on pcDNA3.1(+) backbone, used for transient expression	Generated in this study	Extended Data Fig. 2	1030
GEM17-Halo-FRB	Based on pcDNA3.1(+) backbone, used for transient expression	Generated in this study	Extended Data Fig. 2	1035
GEM22-Halo-FRB	Based on pcDNA3.1(+) backbone, used for transient expression	Generated in this study, available as Addgene #197059	Extended Data Fig. 2	1029
FRB-Halo-GEM23	Based on pcDNA3.1(+) backbone, used for transient expression	Generated in this study	Extended Data Fig. 2	1031
FRB-GEM23-Halo	Based on pcDNA3.1(+) backbone, used for transient expression	Generated in this study, available as Addgene #197060	Extended Data Fig. 2	1032
Halo-FRB	Based on pcDNA3.1(+) backbone, used for transient expression	Generated in this study	Extended Data Fig. 2	1033
IBB-mCherry	Lenti-EF1a_IRES_Blast, used for transient expression	Gift from Daniel W. Gerlich	Extended Data Fig. 6f–h	214
Cas9/sgRNA-AAVS1-1	Based on pGL3 backbone, used for knock-in	Generated in this study	Fig. 1b–d, Fig. 2a–d, i–o, Fig. 3, Fig. 4, Extended Data Fig. 3g,h, 4a,b,d, 5a–c, g–i, 6a–c, i–k,7, 8, 9	642
Cas9/sgRNA-AAVS1-2	Based on pGL3 backbone, used for knock-in	Generated in this study	Fig. 1b–d, Fig. 2a–d, i–o, Fig. 3, Fig. 4, Extended Data Fig. 3g,h, 4a,b,d, 5a–c, g–i, 6a–c,i–k, 7, 8, 9	643
AcGFP-Sec61β	Based on pAcGFP1-C1 backbone, used for transient expression	Addgene #15108	Fig. 5	141
AcGFP-Rab5B	Based on pAcGFP1-C1 backbone, used for transient expression	Addgene #61802	Fig. 5	194
PEX3(1-42)-mEGFP	Based on pEGFP-N1 backbone, used for transient expression	Addgene #120174	Fig. 5	197
LAMP1-mEGFP	Based on pEGFP-N1 backbone, used for transient expression	Addgene #120172	Fig. 5	193
mEGFP-NPM1	Based on pFUGW backbone, used for transient expression	Generated in this study	Fig. 5	443
EGFP-CENP-A	Based on pEGFP-C1 backbone, used for transient expression	Gift from Kevin F. Sullivan	Fig. 5	11
EGFP-G3BP1	Based on pIRESpuro2 backbone, used for transient expression	Generated in this study	Fig. 5, Extended Data Fig. 9	777

Supplementary Table 5: Primers used in this study.

Plasmid	Primer	Sequence (5′–3′)
GEM2-Halo-FRB-IRES-adaptor	TREtight promoter Forward	TGCAAGTGCAGGTGCCAGAACATTTCTCTAGTCGACTCCTCTGCAGAATGC
GEM2-Halo-FRB-IRES-adaptor	TREtight promoter Reverse	AACTAGAAGGCACAGCCAGCACACTGGATCAGTTATCTATG
GEM2-Halo-FRB-IRES-adaptor	bGH polyA Forward	GATCCAGTGTGCTGGCTGTGCCTTCTAGTTGCCAGC
GEM2-Halo-FRB-IRES-adaptor	bGH polyA Reverse	TATCGTCGAACGCGTCCATAGAGCCCACCGCATC
GEM2-Halo-FRB-IRES-adaptor	EF1a promoter-rtTA3 Forward	CGGTGGGCTCTATGGACGCGTTCGACGATAAGC
GEM2-Halo-FRB-IRES-adaptor	EF1a promoter-rtTA3 Reverse	TCCGCTAGTGGATCCCCCGGGGAGCATGTCAAGGTCAAAA
GEM2-Halo-FRB-IRES-adaptor	T2A-Puro-P2A-CD90 Forward	GACATGCTCCCCGGGGGATCCACTAGCGGAAGCG
GEM2-Halo-FRB-IRES-adaptor	T2A-Puro-P2A-CD90 Reverse	GGGGCCGCCCCGGGTGGCTTCGGTCGGAGCACGCGTTCACAGAGAAATGAAGT
GEM2-Halo-FRB-IRES-adaptor	GEM2-Halo-FRB Forward	GGGAGACCCAAGCTTGGTACGCCACCATGGGATCCACCGATAACG
GEM2-Halo-FRB-IRES-adaptor	GEM2-Halo-FRB Reverse	CATGCTCGACCTACTTTGAGATTCGTCGGAAC
GEM2-Halo-FRB-IRES-adaptor	IRES Forward	CTCAAAGTAGGTCGAGCATGCATCTAGGG
GEM2-Halo-FRB-IRES-adaptor	IRES Reverse	GTCGACTCTAGAGGATCCCGGGTTGTGGCAAGCTTATCAT
GEM2-Halo-FRB-IRES-adaptor	Adaptor Forward	ACAACCCGGGATCCTCTAGAGTCGACATGAGAGGAGTGCAGGTGG
GEM2-Halo-FRB-IRES-adaptor	Adaptor Reverse	CACTGGATCAGTTATCTATGCGGCCGCTTAGCTGGAGAC
GEM2-Halo-FKBP	FKBP Forward	GGTAAGCTTGGAAGCGCCGGCTCTGCCGCTGGCAGCGGCGAGGGCGGAGTGCAGGTGGAAACCATCTCC
GEM2-Halo-FKBP	FKBP Reverse	CTGGATATCCTATTCCAGTTTTAGAAGCTCCACATCGAAGACG
GEM2-Halo-FKBP	GEM2-Halo-FKBP Forward	GCTAGCCTCGAGAATTTTGCCACCATGGGATCCACCGA
GEM2-Halo-FKBP	GEM2-Halo-FKBP Reverse	GGAATTGGGCTAGAGCCTATTCCAGTTTTAGAAGCTCCACA
Mito-mCherry-FRB	Mito-mCherry-FRB Forward	TTCGAATTCGCCACCATGAAGAG
Mito-mCherry-FRB	Mito-mCherry-FRB Reverse	AGTGCGGCCGCTTATCAGCCGGCCTGCTTTGA

Supplementary Protocol

The genetically encoded multimeric particle (GEM) tag enables intracellular protein localisation within a distance of 10–25 nm from the surface of the GEM particle in cryo-electron tomograms of human cells. The GEM2 tag is based on an archaeal encapsulin, which forms 25-nm icosahedral particles. GEM2 particles are decorated with Halo-tags for fluorescent labelling and FRB for rapalog ligand-induced coupling to GFP on the protein of interest, via an adaptor protein comprising FKBP, SNAP-tag and an anti-GFP nanobody. The doxycycline-inducible GEM2/adaptor gene cassette can be introduced by transient transfection or as a stable knock-in into the human AAVS1 locus. With the current GEM design, unambiguous identification of the tagged protein is not possible due to the symmetry of the tag and the semi-flexible linker. When using this tag, users should pay attention to time-dependent aggregation of the target protein while optimising for low-background and high-efficiency labelling.

Here we present a series of protocols for I) initial assessment of whether GEMs are an efficient label for a protein of interest; II) generation of a stable GEM/adaptor cell line; III) optimisation of GEM labelling; IV) validation of the cellular phenotype upon labelling; and V) cryo-ET imaging of GEM-labelled subcellular structures. The labelling parameters and validation strategy will be protein- and cell-specific, as proteins vary in abundance and accessibility, which may also depend on cell state and cell type. To illustrate the adjustments required, we describe here our procedure for labelling optimisation, phenotype validation and cryo-ET imaging of the surfactant protein Ki-67 on human mitotic chromosomes. We highlight the main considerations for each experiment. Our analyses based on GEM2-labelling of Mito-GFP, Ki-67, Nup96 and seipin have shown that assessment of the efficiency of GEM labelling and background signal by light microscopy are generally reproduced in cryo-electron tomograms. Therefore, we recommend extensive optimisation and validation by light microscopy before performing the cryo-ET experiment.

Reagents and consumables

- Cell culture medium: Dulbecco's modified medium (DMEM; Gibco, 41965-039) containing 10% (v/v) fetal bovine serum (FBS; Thermo Fisher Scientific, 10270-106), 1% (v/v) penicillin–streptomycin (Gibco, 15140-122), 1 mM sodium pyruvate (Thermo Fisher Scientific, 11360039).
- Imaging medium: FluoroBrite DMEM (Imaging medium; Gibco, A18967-01) containing 10% (v/v) FBS, 1% (v/v) penicillin–streptomycin, 1 x GlutaMax (Thermo Fisher Scientific, 35050061)
- RPMI medium (Thermo Fisher Scientific, 11875093)
- Opti-MEM (Gibco, 11058-021)
- 0.25% (w/v) Trypsin-EDTA (Gibco, 25200-056)
- Puromycin (Merck, 540411)
- EndoFree Plasmid Maxi Kit (Quiagen, 12362)
- Polyethylenimine Max (PEI; Polysciences, 24765)
- Lipofectamine RNAiMAX (Thermo Fisher Scientific, 13778030)
- Halo-TMR (Promega, G8252)
- SNAP-SiR (New England Biolabs, S9102S)
- SiR-DNA (Spirochrome, SC007)
- Hoechst33342 (Thermo Fisher Scientific, H1399)
- Doxycycline (Sigma, D9891)
- (+)-S-Trityl-L-cysteine (STLC; Sigma, 164739)
- Nocodazole (Sigma, M1404)
- Thymidine (Sigma, T1895)
- 0.5 mM A/C Heterodimerizer (Rapalog; TAKARA, 635056)
- Phosphate-buffered saline (PBS), pH 7.4
- 37% formaldehyde (Merck, 104003)
- FACS buffer: 2% FBS and 2 mM EDTA in PBS
- 0.2 mg/mL APC anti-rat CD90/mouse CD 90.1 (BioLegend, 202526)
- Fibronectin (Sigma, F1141)
- Trypan Blue Solution, 0.4% (Thermo Fisher Scientific, 15250061)
- Gold or titanium grids with holey SiO_2 R1.2/20 film, 200 mesh (Quantifoil)
- µ-Slide 8 Well high Glass Bottom (ibidi, 80807)
- µ-Dish 35 mm, low (ibidi, 80136)
- LabTek 8-well chambered coverglass (Thermo Fisher Scientific, 155411)
- 96-well imaging plates (MoBiTec, 5241-20)
- Countess Cell Counting Chamber Slides (Thermo Fisher Scientific, C10228)
- Neon Transfection System (Thermo Fisher Scientific, MPK5000)
- Neon Transfection System Pipette (Thermo Fisher Scientific, MPP100)
- Neon Transfection System 100 µL Kit (Thermo Fisher Scientific, MPK10025)
- Test Tube with Cell Strainer Snap Cap 35 µm mesh (Corning, 352235)

Equipment

- Cell counter, e.g. Thermo Fisher Scientific Countess 3 Automated Cell Counter
- Confocal microscope, e.g. Zeiss LSM 780 with EC Plan-Neofluar 40×/1.30 Oil DIC M27 oil-immersion objective, Zeiss LSM 980 with Airyscan2 and Plan-Apochromat 63×/1.4 Oil DIC M27 objective
- Widefield microscope, e.g. Molecular Devices ImageXpress Micro with CFI Plan Apochromat Lambda 4×/0.20 and 20×/0.75 objectives
- Plunge freezer, e.g. Leica EM GP2
- Optional: Cryo-fluorescence microscope, e.g. Zeiss LSM 900 Airyscan2 microscope with a Linkam cryo-stage
- Cryo-focused ion beam/scanning electron microscope, e.g. Thermo Fisher Scientific Aquilos
- 300 kV transmission electron microscope with an energy filter and direct electron detector, e.g. Thermo Fisher Scientific Titan Krios

Software

- CellProfiler[67] (open source, https://cellprofiler.org/)
- Fiji[68] (open source, https://imagej.net/software/fiji/)
- ilastik[66] (open source, https://www.ilastik.org/)
- Icy (open source, https://icy.bioimageanalysis.org/)

I. Testing GEM labelling of GFP-tagged protein of interest

Here we provide a general protocol to determine if GEMs can label a GFP-tagged protein of interest efficiently. The protein of interest may be endogenously tagged (via CRISPR/Cas9 gene editing[90]), stably overexpressed (via random integration, viral vector-based gene transfer) or transiently overexpressed. Prior to the experiment, it is important to verify that GFP-tagging itself does not perturb protein function or localisation. For an initial test of GEM labelling, we recommend transient transfection of the GEM2/adaptor expression plasmid and variation of labelling time (rapalog-induced coupling of GEM2) as described below. This allows for quick assessment of GEM expression, particle formation, and binding of adaptor protein to the GFP-tagged protein of interest. If co-localisation between GEMs and the GFP-tagged protein upon rapalog treatment is observed, we recommend generation of a stable cell line (see Section II).

GEM2/adaptor plasmid transfection

1. Prepare transfection mix containing the GEM2/adaptor expression plasmid (Addgene #197061) in a 1.5 mL tube. Incubate mix at RT for 15 min.

Reagent	Amount
Opti-MEM	50 µL
Plasmid DNA	1 µg
PEI	3 µL

2. Optional: If transient transfection with the GFP-tagged protein is required, add 1 µg expression plasmid and 3 µL PEI to the above transfection mix.
3. Seed cells in an ibidi 8-well µ-Slide, in a total volume of 400 µL medium, to reach 80% confluency in 2 days. Allow for a range of rapalog treatment times, as labelling kinetics can vary for each protein. To assess the leakiness of the TREtight promoter, include one well where doxycycline is not added. An example scheme is provided below.

Well	Transfection	Doxycycline Treatment	Rapalog Treatment
1	−	−	−
2	+	−	−
3	+	+	−
4	+	+	0.5 h
5	+	+	1 h
6	+	+	2 h
7	+	+	4 h
8	+	+	8 h

4. Add 25 µL transfection mix per transfection (+) well. Incubate for 24 h.

Doxycycline induction and rapalog treatment

5. Add 1 µg/mL doxycycline to the doxycycline (+) samples. Incubate for 18–24 h.
6. Replace medium with 300 µL fresh medium containing 1 µg/mL doxycycline.
7. Add 0.5 µM rapalog to the appropriate wells, e.g. by preparing a 5x rapalog solution (2.5 µM) in doxycycline containing medium and adding 75 µL to each well. Incubate for 0.5 h, 1 h, 2 h, 4 h, and 8 h rapalog treatment before proceeding with fixation.

Cell fixation and staining

8. Wash cells 3 times with PBS. Fix cells with 3.7% formaldehyde in PBS at RT for 15 min.
9. Wash cells 3 times with PBS. Label GEMs and adaptor through their Halo-tag and SNAP-tag, e.g. with 100 nM Halo-TMR and 100 nM SNAP-SiR, according to the manufacturer's recommendations. While staining of adaptor protein can be omitted in subsequent tests, at this stage, it is important to verify binding of the adaptor protein to the GFP-tagged protein.
10. Wash cells 3 times with PBS. Optional: stain DNA with 0.2 µg/mL Hoechst33342 in PBS at RT for 15 min.
11. Wash cells twice with PBS. Keep cells in PBS at 4 °C. Protect from light.

Imaging and image analysis

12. Image GFP, GEM and adaptor protein fluorescence on a confocal microscope with high NA objective, e.g., Zeiss LSM780 with Plan-Apochromat 63x/1.4 Oil DIC M27. Depending on the protein of interest, it may be useful to acquire z-stacks.
13. Doxycycline treatment induces expression of GEM and adaptor protein. Examine images upon doxycycline treatment for punctate GEM fluorescence and colocalisation of adaptor protein with GFP. Rapalog treatment induces coupling of GEMs to the adaptor protein. Therefore, colocalisation of GEMs with adaptor protein and GFP should be observed upon rapalog treatment. Take note of any changes in protein localisation upon GEM labelling, especially at longer treatment times. This will help set an upper limit for rapalog treatment time during labelling optimisation.

II. Generating a stable GEM2/adaptor knock-in cell line

While it is possible to perform cryo-ET imaging on cells transiently transfected with GEM2/adaptor plasmid, we recommend generating a stable cell line where GEM2 and adaptor protein are expressed from the AAVS1 safe-harbour locus. This gives better reproducibility, more control over GEM2 expression levels and prevents additional cellular stress prior to cell vitrification. Generation of the stable cell line can be performed in a GFP-fusion background for the protein of interest or in wildtype cells.

<u>Cell electroporation and cell selection</u>

1. Mix 5 µg Cas9/gRNA-1 plasmid (Addgene #129726), 5 µg Cas9/gRNA-2 plasmid (Addgene #129727), and 7.5 µg GEM2/adaptor donor plasmid (Addgene #197061) in a 1.5 mL tube.
2. Suspend 2.0×10^6 cells in 120 µL Buffer R of the Neon Transfection System.
3. Add 100 µL cell suspension to the plasmid mix.
4. Draw 100 µL of the cell and plasmid mix into a 100 µL Neon tip.
5. Electroporate as optimised for the cell line used, e.g. 1300 V, 10 ms pulses, 3 times for Hela cells.
6. Transfer cells into 0.5 mL RPMI medium containing 10% FBS in a 1.5 mL tube.
7. Incubate at 37 ºC for 15 min.
8. Transfer cells to a 10 cm dish with 10 mL medium, no penicillin-streptomycin. Culture for 1 day.
9. Replace the medium with the fresh medium with penicillin-streptomycin. Culture for 1 day.
10. Select cells with 0.5 µg/mL puromycin, exchanging medium every 2–3 days. Treat one dish of non-transfected cells as a control. For HeLa cells, non-transfected cells typically die over 2–3 days whereas transfected cells recover within 1 week. After 1 week, typically 60–70% of cells express the constitutive knock-in selection marker CD90.1.
11. Dilute to single clones in a 96-well plate. Optional: Fluorescence-activated cell sorting can be performed to enrich for successful knock-in. Sort live cells positive for CD90.1 (via antibody staining) into 96-well plates. Cells can also be sorted for GEM expression following doxycycline treatment and Halo labelling.

<u>Microscopy validation of monoclonal cells</u>

12. Identify candidate colonies with uniform cell size within the colony by brightfield imaging, e.g. with a high-content imaging system such as a Molecular Devices ImageXpress Micro with CFI Plan Apochromat Lambda 4×/0.20 objective. Candidate colonies can be identified in a semi-automated manner, e.g. with the PlateViewer plugin[91] in Fiji.
13. For each candidate colony, transfer cells to a 96-well plate for further subculture and to two 96-well imaging plates for fluorescence imaging.
14. Add 1 µg/mL doxycycline to one imaging plate and incubate for 18–24 h.
15. Replace medium in both imaging plates with 100 µL fresh medium containing 50 nM Halo-TMR and 0.2 µg/mL Hoechst33342. Incubate at 37 ºC for 30 min.
16. Wash both imaging plates 3 times with pre-warmed PBS. Replace with 100 µL imaging medium.
17. Record Halo-TMR fluorescence, using Hoechst33342 signal for focusing, e.g. with a Molecular Devices ImageXpress Micro with CFI Plan Apochromat Lambda 20×/0.75 objective.
18. Select clones with high Halo-TMR fluorescence in the doxycycline-treated plate and no fluorescence in the untreated plate. Expand clones.
19. Verify GEM labelling. Select clones that show the most efficient labelling.

III. Optimization of GEM2 labelling duration

GEM2 labelling duration should be kept as brief as possible, but still long enough to label a significant fraction of the target GFP-tagged protein. This optimal labelling duration is protein- and cell-specific. We recommend performing optimisation in the cell line that will be used for cryo-ET imaging. As stated in the previous section, we recommend knocking in the GEM2/adaptor cassette. We illustrate the protocol here with Ki-67. To observe Ki-67 on mitotic chromosomes, cells were synchronized in early mitosis by a single thymidine block followed by STLC arrest as outlined below. During synchronisation, GEM2/adaptor expression is induced. Upon release from arrest, different GEM2 labelling (rapalog treatment) times are tested. The best labelling time is determined by analysing the overlap between GEM2 and Ki-67 fluorescence using image analysis software ilastik and CellProfiler. Adaptation will be necessary for each new protein of interest.

<u>Cell cycle synchronisation and doxycycline induction.</u>

1. Seed cells expressing endogenously tagged EGFP-Ki-67 with AAVS1 GEM2/adaptor knock-in into an 8-well LabTek chambered coverglass (25,000 cells/well).
2. Add 2 µg/mL doxycycline and 2 mM thymidine. Incubate for 24 h.
3. Wash 3 times with pre-warmed fresh medium. Replace with 400 µL medium containing 10 µM STLC and 2 µg/mL doxycycline. Incubate for 24 h.

<u>Live staining and rapalog treatment</u>

4. Replace medium with 250 µL medium containing 100 nM Halo-TMR, 10 µM STLC and 2 µg/mL doxycycline. Incubate at 37 °C for 20 min.
5. Wash 3 times with pre-warmed fresh medium. Replace with 400 µL medium. Add 0.5 µM rapalog. Incubate for 15, 30 and 60 min. These timepoints will depend on the target and should be based on preliminary results obtained in section I.

<u>Cell fixation</u>

6. Wash twice with PBS. Fix cells with 3.7% formaldehyde in PBS at RT for 15 min.
7. Wash 3 times with PBS. Stain DNA with 0.2 µg/mL Hoechst33342 in PBS at RT for 15 min.
8. Wash twice with PBS. Keep cells in PBS at 4 °C until imaging. Protect from light.

<u>Imaging and image analysis</u>

9. Image fixed cells, e.g., on a Zeiss LSM980 confocal microscope, equipped with an Airyscan detector and Plan-Apochromat 63×/1.4 Oil DIC M27 objective. While any confocal microscope can be used, Airyscan imaging and processing enable more robust segmentation of GEMs by fluorescence.
10. Acquire GFP, DNA and GEM fluorescence images at a single plane.
11. Segment cell boundaries manually and measure the cell area in Fiji.
12. Segment GEMs and Ki-67 using ilastik, train in Pixel Classification mode using 5 images from each rapalog time point. Perform batch pixel classification to generate probability maps. Threshold GEM and Ki-67 probability maps at 0.5 in CellProfiler.
13. Measure the percentage of GEM area overlapping with Ki-67 and the percentage of Ki-67 overlapping with GEM area for each cell using CellProfiler, and plot the results for each time point. This yields an overall assessment of GEM labelling efficiency at different time points. In addition, measure relative

GEM abundance per cell by dividing the number of individual GEM objects in a cell by the cell area. For each cell, plot the percentage of GEM area overlapping with Ki-67 vs relative GEM abundance. Similarly, for each cell, plot the percentage of Ki-67 area overlapping with GEMs vs relative GEM abundance. These analyses help to decipher which level of GEM abundance is best suited to give maximal target coverage but still an acceptable level of unbound GEM background.

IV. Assessment of cellular phenotype upon GEM2 labelling

The binding of GFP nanobodies[92] and GEMs can interfere with protein function or localisation. Therefore, we recommend assessing the phenotype of cells upon GEM2 labelling within the timeframe identified in III. For Ki-67, loss of function in early mitosis is indicated by coalescence of mitotic chromosomes[36], detectable as a reduction in chromosome ensemble area in nocodazole-arrested cells upon DNA staining. We suggest comparing between cells not expressing GEMs or adaptor protein, cells expressing GEMs and adaptor protein with and without rapalog treatment, and cells depleted of the protein of interest, via knockout or siRNA-mediated knockdown.

Cell seeding, doxycycline induction, live staining and nocodazole arrest

1. Seed cells expressing endogenously tagged EGFP-Ki-67 with AAVS1 GEM2/adaptor knock-in into an 8-well LabTek chambered coverglass (25,000 cells/well). Seed Ki-67 knockout cells as a control.
2. Optional: Seed cells expressing endogenously tagged EGFP-Ki-67 for siRNA-mediated knockdown (20,000 cells/well). Transfect negative control siRNA and Ki-67 siRNA[36] with Lipofectamine RNAiMAX according to the manufacturer’s protocol. Incubate for 48 h.
3. Add 2 µg/mL doxycycline. Incubate for 24 h.
4. Replace culture medium with 250 µL medium containing 2 µg/mL doxycycline and 100 nM Halo-TMR. Incubate at 37 °C for 20 min.
5. Wash with fresh medium 3 times. Replace with 400 µL imaging medium containing 2 µg/mL doxycycline, 0.2 µM SiR-DNA and 0.2 µg/mL nocodazole. Incubate for 2 h.

Live cell imaging

6. Image cells on a confocal microscope, e.g., a Zeiss LSM780 with an oil-immersion objective. Acquire transmission images and z-stacks of DNA and GEM2 fluorescence for EGFP-Ki-67 cells and Ki-67 knock-out cells covering the full height of cells in the field of view (= 0 min rapalog treatment sample).
7. Add 0.5 µM rapalog and 100 nM Halo-TMR.
8. Acquire images after 15, 30 and 60 min of rapalog treatment.

Measuring ensemble chromosome area

9. Crop single cells manually in Fiji based on DNA signal and transmission image.
10. Select a single slice for subsequent analysis based on the highest DNA mean intensity across slices.
11. Segment whole chromosome regions in ilastik using the DNA signal. Train in Pixel Classification mode using 20 images and perform batch pixel classification to generate probability maps. Then train in Object Classification mode using 10 images. Classify into mitotic chromosomes and small nonspecific staining. Perform batch analysis of all images using the trained dataset.
12. Analyse the ensemble chromosome area per cell in the different samples. A consistent chromosome area compared to time zero suggests no gross perturbation of protein function upon GEM2 labelling, whereas Ki-67 knockdown is expected to cause chromosome area reduction.
13. For each cell, also plot the whole chromosome area against mean GEM intensity at chromosomes to compare chromosome size alterations between cells exhibiting high and low GEM2 labelling. The lack of a correlation between chromosome area and GEM intensity further implies no gross perturbation of protein function upon GEM2 labelling.

V. Cryo-ET sample preparation, imaging and analysis

Once optimised, GEM labelling can be performed in cells seeded on an EM grid, followed by vitrification at the appropriate rapalog incubation time. Specific to the Ki-67 experiment are the selection of mitotic cells for FIB milling by fluorescence imaging prior to freezing, and cryo-Airyscan imaging of the final lamellae to delineate the location of mitotic chromosome boundaries for cryo-ET data acquisition, as detailed here. Further selection of cells based on GEM fluorescence levels before or after freezing to fine-tune labelling specificity as described is also possible here.

Micropatterning of EM grids

1. Optional: Micropattern EM grids to optimally position cells in grid squares to increase the number of usable areas per grid. Micropattern as described by Toro-Nahuelpan et al.[72] to produce 8 × 8 30-µm-diameter PEG-free circles, each in the centre of one grid square, in the central region of the grid. Briefly, passivate grids with PLL-g-PEG, irradiate circular patterns with a UV laser, e.g., on a Nikon Eclipse Ti and Alveole Primo micropatterning system. Store grids in PBS on Parafilm at 4 °C until use.

Cell cycle synchronisation by double thymidine block

2. Seed cells expressing endogenously tagged EGFP-Ki-67 with GEM2/adaptor AAVS1 locus knock-in in a 6 cm dish (2.0 × 10^5 cells/dish).
3. Add 2 µg/mL doxycycline and 2 mM thymidine and incubate for 24 h.
4. Wash 3 times with fresh medium to release cells from arrest.
5. Replace with fresh medium supplemented with 2 µg/mL doxycycline. Incubate for 8 h.
6. Add 2 mM thymidine and incubate for 14–20 h.

Cell seeding on grids, release from arrest and live staining

7. Coat micropatterned EM grids in 10–15 µL drops of 1 mg/mL fibronectin at RT for 30 min.
8. Wash 3 times in PBS and place film-side-up in a 35-mm ibidi low µ-Dish with 1 mL fresh medium.
9. Wash cells with pre-warmed medium 3 times. Trypsinize with trypsin-EDTA at 37 °C for 5 min. Resuspend with fresh medium. Pellet cells at RT, 90 × g, 3 min. Resuspend in fresh medium and pellet again. Resuspend cells in 2 mL fresh medium.
10. Optional: Pass cells through a 70-µm cell strainer to remove cell clumps.
11. Add 2 × 10^5 cells dropwise over grids. Incubate at 37 °C for 1 h until cells have attached on the grids.
12. Prepare a new 35-mm low µ-Dish with 1 mL fresh medium containing 2 µg/mL doxycycline. Prepare another 6-cm dish with 4 mL fresh medium for rinsing.
13. Pass grids through the 6-cm dish and transfer to the new 35 mm dish. Incubate for 4 h.
14. Aspirate and replace with fresh medium containing 2 µg/mL doxycycline and 100 nM Halo-TMR. Incubate for 20 min.
15. Wash with fresh medium 3 times. Aspirate and replace with 800 µl fresh medium containing 2 µg/mL doxycycline and 0.2 µM SiR-DNA. Incubate for 4 h. Optional: To arrest cells in mitosis, add nocodazole to a final concentration of 0.2 µg/mL.

Vitrification and cryo-FIB lamella preparation

16. Monitor progress into mitosis on a confocal microscope such as Zeiss LSM780 from 8.5 h post release. Once over 30% of cells enter into mitosis, as indicated by rounding of cells and the emergence of condensed chromosomes, add 0.5 μM rapalog and 0.2 μM SiR-DNA. Incubate for 30–60 min.
17. Optional: Acquire tiled z-stacks of DNA and GEM fluorescence, and in brightfield, to help identify which cells on the grid are mitotic in the cryo-FIB-SEM microscope. Selection of cells with a suitable GEM2 expression level is also possible here.
18. Freeze cells, e.g., with a Leica EM GP2 plunger. Add 3 μL medium to the cell side of grids before blotting to reduce cell flattening. Blot from the back for 1–3 s at 37 °C, 90% humidity, and plunge into liquid ethane at −185 °C. Store grids in liquid nitrogen in well-sealed grid boxes. Clip grids into cryo-FIB auto-grids.
19. Prepare cryo-FIB lamellae, e.g., with a TFS Aquilos FIB-SEM microscope. The details of microscope operation and lamella preparation have been described extensively by Wagner et al.[93]. Optional: Automated FIB milling can be performed using the open-source SerialFIB, described in Klumpe et al.[8] Below are steps we employed to produce lamellae successfully for this sample.
    - Before milling, sputter-coat with inorganic platinum (1 kV, 10 mA, 10 Pa, 15–20 s). Deposit organometallic platinum via the Gas Injection System (GIS, at 28 °C, 10.6 mm working distance, 8–11 s).
    - Mill cells to 1-μm thickness at 20° stage tilt (for a 45°-pretilt shuttle) with decreasing ion beam currents (1, 0.5, 0.3 nA, 30 keV). Fine mill all positions in the final stage together to a target thickness of 200–250 nm at 50 and 30 pA ion beam currents.
    - Optional: Thin the back of lamellae at a stage tilt of 21-22°.
    - Optional: Sputter-coat with inorganic platinum (1 kV, 10 mA, 10 Pa, 5–15 s, target 5 nm layer thickness) to reduce charging and beam-induced motion during TEM imaging.
    - Assess lamellae by scanning electron microscopy (10 kV, 50 pA).

Cryo-Airyscan imaging of FIB lamellae

20. Take an overview widefield image with the 5× air objective to locate lamellae. The specifics of cryo-Airyscan and Linkam stage operation are detailed in Wu et al.[94].
21. Acquire z-stacks in Airyscan mode with the 100× objective, e.g., with 0.5 μm spacing over 4–6 μm at 79 nm pixel size. Record reflection images for each z-plane. Optional: Average 4 times between frames to increase signal-to-noise ratio.
22. Airyscan-process in 2D mode. Generate maximum intensity projections.
23. Register reflection images against low magnification TEM images of lamellae (lamella maps), e.g., using Icy eC-CLEM[73] or Fiji BigWarp[74], with lamella outline and features present on the lamella such as lipid droplets in the reflection images as landmarks.

Cryo-ET imaging and analysis

24. Collect dose-symmetric tilt series as described by Turoňová et al.[95].
25. Align tilt series, e.g. using patch tracking in Etomo[78] or AreTomo[79]. Reconstruct tomograms by weighted backprojection. A good quality alignment is imperative for robust detection of GEMs.
26. Visually inspect tomograms for GEMs. For visualisation purposes, filter or denoise tomograms as needed. Optional: Apply a dedicated CNN to detect GEMs. A DeePiCt model[12] trained on Gatan K2 and K3 defocus and Volta phase plate data is available at https://github.com/hermankhfung. Visually inspect high probability scoring peaks.

Troubleshooting

Problem	Solution
GEM2 and adaptor protein fluorescence detected in the absence of doxycycline.	Check cell culture medium composition. Use tetracycline-free serum.
GEM2 and adaptor protein fluorescence not detected after doxycycline treatment.	Adjust doxycycline treatment time and concentration. Verify transfection protocol using other plasmids.
GEM2 fluorescence diffuse and not punctate	Ensure Halo-tag labelling reaction is done according to protocol. Ensure robust expression of GEM2 (suggested range 12–24 h doxycycline treatment, doxycycline concentration may need adjustments depending on cell line). To properly detect fluorescence puncta, a confocal microscope with a high NA objective is recommended. Some GEM2 background is expected.
Adaptor protein not co-localising with target protein	Verify GFP construct design. Ensure GFP tag is accessible in the cytoplasm or nucleoplasm.
GEM2 does not colocalize with adaptor protein or target protein after rapalog treatment.	Prolong rapalog treatment time.
Changes in target protein localisation or cellular phenotypes upon GEM2-labelling of target	Reduce rapalog and/or doxycycline treatment time and/or concentration. If the function or localisation of target is still affected, reconsider the location of the GFP tag or whether this system would be useful.
Unbound GEM background after rapalog treatment	Optimise GEM2 expression levels or choose cells with appropriate levels, and adjust rapalog treatment times. Utilise on-lamella CLEM to detect specific labelling events based on colocalisation of GEM and target fluorescence. Some unbound GEMs are expected. For Ki-67, a small fraction of the protein is also present as a soluble cytosolic pool, which can give rise to an apparent background.
No detectable GEM particles in tomograms	Verify GEM expression and labelling by light microscopy. Screen for GEM-expressing cells on the EM grid based on fluorescence. For unambiguous GEM2 detection, good contrast in the tomogram is essential. Ensure lamella is sufficiently thin (< 250 nm), exposure dose, camera dose rate are appropriate, sample is not charging, stage is stable, tilt series alignment is accurate.
DeePiCt CNN fails to identify GEMs	Check tomograms are pre-processed consistently with the DeePiCt model. If data are significantly different from the training set used, it may be necessary to train a new CNN model to ensure robust detection. Always validate visually that particles identified are GEM-like (25-nm size, icosahedral shape). Subtomogram averaging (and classification if a large dataset is available) of detected GEM-particles can assist with verification.

References

90. Koch, B. *et al.* Generation and validation of homozygous fluorescent knock-in cells using CRISPR–Cas9 genome editing. *Nat. Protoc.* **13**, 1465–1487 (2018).
91. Besson, S. & Tischer, C. embl-cba/fiji-plugin-plateViewer: PlateViewer: Fiji plugin for visual inspection of high-throughput microscopy based image data. *Zenodo* (2019) doi:10.5281/zenedo.3522688.
92. Küey, C., Larocque, G., Clarke, N. I. & Royle, S. J. Unintended perturbation of protein function using GFP nanobodies in human cells. *J. Cell Sci.* **132**, (2019).
93. Wagner, F. R. *et al.* Preparing samples from whole cells using focused-ion-beam milling for cryo-electron tomography. *Nat. Protoc.* **15**, 2041–2070 (2020).
94. Wu, G. H. *et al.* Multi-scale 3D cryo-correlative microscopy for vitrified cells. *Structure* **28**, 1231-1237.e3 (2020).
95. Turoňová, B. *et al.* Benchmarking tomographic acquisition schemes for high-resolution structural biology. *Nat. Commun.* **11**, 1–9 (2020).
