# Supplementary material for: Genetically encoded multimeric tags for subcellular protein localization in cryo-EM
Source: Nat Methods. 2023 Nov 6;20(12):1900–8. doi: 10.1038/s41592-023-02053-0 (PMC10703698; doi:10.1038/s41592-023-02053-0)
Supplement: Supplementary file 2 — Reporting Summary [file 41592_2023_2053_MOESM2_ESM.pdf]

nature portfolio

Corresponding author(s): Julia Mahamid

Last updated by author(s): 24/08/2023

Reporting Summary

Nature Portfolio wishes to improve the reproducibility of the work that we publish. This form provides structure for consistency and transparency in reporting. For further information on Nature Portfolio policies, see our Editorial Policies and the Editorial Policy Checklist.

Statistics

For all statistical analyses, confirm that the following items are present in the figure legend, table legend, main text, or Methods section.

n/a	Confirmed	
☐	☒	The exact sample size (*n*) for each experimental group/condition, given as a discrete number and unit of measurement
☐	☒	A statement on whether measurements were taken from distinct samples or whether the same sample was measured repeatedly
☐	☒	The statistical test(s) used AND whether they are one- or two-sided<br>*Only common tests should be described solely by name; describe more complex techniques in the Methods section.*
☒	☐	A description of all covariates tested
☐	☒	A description of any assumptions or corrections, such as tests of normality and adjustment for multiple comparisons
☐	☒	A full description of the statistical parameters including central tendency (e.g. means) or other basic estimates (e.g. regression coefficient) AND variation (e.g. standard deviation) or associated estimates of uncertainty (e.g. confidence intervals)
☐	☒	For null hypothesis testing, the test statistic (e.g. *F*, *t*, *r*) with confidence intervals, effect sizes, degrees of freedom and *P* value noted<br>*Give P values as exact values whenever suitable.*
☒	☐	For Bayesian analysis, information on the choice of priors and Markov chain Monte Carlo settings
☒	☐	For hierarchical and complex designs, identification of the appropriate level for tests and full reporting of outcomes
☒	☐	Estimates of effect sizes (e.g. Cohen's *d*, Pearson's *r*), indicating how they were calculated

Our web collection on statistics for biologists contains articles on many of the points above.

Software and code

Policy information about availability of computer code

Data collection

Fluorescence microscopy
- Zeiss LSM780 operated with ZEN 2.3 (black)
- Zeiss LSM880 operated with ZEN 2.1 (black)
- Zeiss LSM980 Airyscan2 and LSM900 Airyscan2 operated with ZEN 3.4 (blue)
- Zeiss Axio Observer operated with ZEN 2.3 (blue)
- Olympus IXplore SpinSR operated with cellSens Dimension 3.2

EM grid micropatterning
- Nikon Eclipse Ti and Alveole Primo micropatterning system operated with Leonardo 4.16 through µManager 1.4.22 (doi: 10.14440/jbm.2014.36)

Cryo-FIB milling
- Thermo Fisher Scientific Aquilos operated with SerialFIB 1.0 (github.com/sklumpe/SerialFIB)

Cryo-electron tomography
- Thermo Fisher Scientific Krios operated with SerialEM 4.1.0beta (doi: 10.1016/j.jsb.2005.07.007)

Data analysis

Fluorescence image analysis
- Fiji v2.3.0/1.53t (doi: 10.1038/nmeth.2019)
- CellProfiler 1.0.5122 (doi: 10.1186/gb-2006-7-10-r100), used for analysis in Extended Data Fig 6i-k.
- CellProfiler 4.1.3 (doi: 10.1186/s12859-021-04344-9)

- ilastik v1.4.0rc5 (doi: 10.1038/s41592-019-0582-9)
- ObjectAnalyser v0 (bitbucket.org/szkabel/lipidanalyser/get/master.zip)
- FRAPAnalyser v2.1.0 (github.com/ssgpers/FRAPAnalyser)
- FCSRunner v0.8.1 (git.embl.de/grp-ellenberg/fcsrunner)
- MyPic v0.8.3 (git.embl.de/grp-ellenberg/mypic)
- Fluctuation Analyzer 4G v 15.02.23 (http://www.fluctuations.de)
- FCSFitM v0.9 (git.embl.de/grp-ellenberg/FCSAnalyze)
- FCSImageBrowser v0.4.3_0.9 (git.embl.de/grp-ellenberg/FCSAnalyze)
- FCSCalibration v0.4.3 (git.embl.de/grp-ellenberg/FCSAnalyze)

Numerical data analysis and visualisation
- Python 3.8.12
- Microsoft Excel 16.67
- MathWorks MATLAB 2020a
- RStudio 1.4.1717, R version 4.1.0 (www.rstudio.com)
- Gnuplot 5.4 (gnuplot.sourceforge.net)
- GraphPad Prism 9.3.1

Phylogenetic analysis and visualisation
- MAFFT 7.490 (doi: 10.1093/molbev/mst010)
- BMGE 1.12 (doi: 10.1186/1471-2148-10-210)
- SMS 2.0 (doi: 10.1093/molbev/msx149)
- PhyML 3.3.20190321 (doi:10.1093/sysbio/syq010)
- iTol v6 (doi: 10.1093/nar/gkab301)

Cryo-electron tomography
- Icy eC-CLEM plugin 1.0.1.5 (doi: 10.1038/nmeth.4170)
- Warp 1.0.9 (doi: 10.1038/s41592-019-0580-y)
- AreTomo 1.3.1 (doi: 10.1016/j.yjsbx.2022.100068)
- IMOD 4.11.12 (doi: 10.1006/jsbi.1996.0013)
- EMAN2 2.91 (doi: 10.1016/j.jsb.2006.05.009)
- DeePiCt 1.0.0 (github.com/ZauggGroup/DeePiCt)
- DeePiCt model for automated GEM detection (github.com/hermankhfung/GEM)
- Python script for GEM-target distance analyses (github.com/hermankhfung/GEM)
- RELION 4.0.0-beta-2 (doi: 10.1042/BCJ20210708)
- TomoSegMemTV April 2020 (doi: 10.1016/j.jsb.2014.02.015)
- Thermo Fisher Scientific Amira 2022.1
- ChimeraX 1.4 (doi: 10.1002/pro.3943)

For manuscripts utilizing custom algorithms or software that are central to the research but not yet described in published literature, software must be made available to editors and reviewers. We strongly encourage code deposition in a community repository (e.g. GitHub). See the Nature Portfolio guidelines for submitting code & software for further information.

Data

Policy information about availability of data

All manuscripts must include a data availability statement. This statement should provide the following information, where applicable:

- Accession codes, unique identifiers, or web links for publicly available datasets
- A description of any restrictions on data availability
- For clinical datasets or third party data, please ensure that the statement adheres to our policy

The subtomogram average of GEM2 is available on EMDB under entry EMD-16303. Cryo-ET data for GEM labelling of Mito-EGFP (including raw data, tilt-series, reconstructed tomograms and GEM coordinates) are deposited on EMPIAR under entry EMPIAR-11561. A representative tomogram is deposited on EMDB under entry EMD-18194. The atomic model of the S. elongatus encapsulin scaffold was obtained from the Protein Data Bank (PDB 6X8M).

Human research participants

Policy information about studies involving human research participants and Sex and Gender in Research.

Reporting on sex and gender	No human research participants were involved in this study.
Population characteristics	N/A
Recruitment	N/A
Ethics oversight	N/A

Note that full information on the approval of the study protocol must also be provided in the manuscript.

Field-specific reporting

Please select the one below that is the best fit for your research. If you are not sure, read the appropriate sections before making your selection.

☒ Life sciences ☐ Behavioural & social sciences ☐ Ecological, evolutionary & environmental sciences

For a reference copy of the document with all sections, see nature.com/documents/nr-reporting-summary-flat.pdf

Life sciences study design

All studies must disclose on these points even when the disclosure is negative.

Sample size	No sample-size calculations were performed. Sample sizes were chosen to be as large as possible while taking into account the experimental effort and resources required to generate the respective data. Adequate statistics has been applied throughout the manuscript to ensure the observed effects are significant given the reported sample size.
Data exclusions	Cells not completely in the field of view in fluorescence images were excluded from analysis. Tomograms of poor reconstruction quality or contrast (discontinuous or weak membrane densities, weak ribosomal densities), typically originating from FIB-lamellae regions thicker than 300 nm, were not used for neural network training or subtomogram averaging. DeePiCt GEM particle predictions were manually verified. Clear mismatches, e.g., ribosomes, were excluded from subsequent rounds of neural network training and subtomogram averaging.
Replication	All fluorescence microscopy experiments were performed at least twice; data were pooled for analysis. Cryo-electron tomography data were acquired from multiple grids of cells grown and frozen on different days: Mito, 3 freezing sessions, 6 grids, 12 lamellae, 17 tomograms; Ki-67, 2 freezing sessions, 3 grids, 3 lamellae, 9 tomograms; Nup96, 1 freezing session, 2 grids, 6 lamellae, 20 tomograms; seipin, 4 freezing sessions, 5 grids, 9 lamellae, 24 tomograms. All attempts at replication were successful.
Randomization	Randomization was not performed. Data were pooled per sample type (a specific protein tagged with GFP) and not further allocated into subgroups.
Blinding	Blinding was not performed. Analysis procedures were tailored per sample type and applied uniformly across all data of the same sample type.

Reporting for specific materials, systems and methods

We require information from authors about some types of materials, experimental systems and methods used in many studies. Here, indicate whether each material, system or method listed is relevant to your study. If you are not sure if a list item applies to your research, read the appropriate section before selecting a response.

Materials & experimental systems

n/a	Involved in the study	
☐	☒	Antibodies
☐	☒	Eukaryotic cell lines
☒	☐	Palaeontology and archaeology
☒	☐	Animals and other organisms
☒	☐	Clinical data
☒	☐	Dual use research of concern

Methods

n/a	Involved in the study	
☒	☐	ChIP-seq
☒	☐	Flow cytometry
☒	☐	MRI-based neuroimaging

Antibodies

Antibodies used	for FACS sorting: APC anti-rat CD90/mouse CD90.1 (Thy-1.1), Biolegend, catalog number 202526, clone OX-7, lot B236123
Validation	APC anti-rat CD90/mouse CD90.1 (Thy-1.1) was validated in Zhang Y, et al., Enhancing CD8+ T Cell Fatty Acid Catabolism within a Metabolically Challenging Tumor Microenvironment Increases the Efficacy of Melanoma Immunotherapy. Cancer Cell. 11;32(3):377-391.e9. (2017), doi: 10.1016/j.ccell.2017.08.004.

Eukaryotic cell lines

Policy information about cell lines and Sex and Gender in Research

Cell line source(s)	- All HeLa cell lines were derived from a HeLa Kyoto cell line obtained from S. Narumiya (Kyoto University, Japan); RRID:CVCL_1922; human; female.<br>- U-2 OS-CRISPR-NUP96-mEGFP clone no.195; RRID:CVCL_B7FJ; human; femal (CLS Cell Lines Services GmbH, Eppelheim, Germany).

	- SUM159 cells were a gift from the lab of R. Farese and T. Walther (Sloan Kettering Institute, USA); RRID:CVCL_5423; human; female.
Authentication	The wild-type HeLa Kyoto cell line, from which other HeLa cell lines in this study were derived, were validated by a Multiplex human cell line Authentication test (MCA), 21.04.2016. The remaining cell lines used were not authenticated.
Mycoplasma contamination	PCR-based mycoplasma tests were performed every 3 to 6 months and were negative for all cell lines.
Commonly misidentified lines (See ICLAC register)	No common misidentified cell lines were used in this study.
